# *Sporobolus stapfianus:* Insights into desiccation tolerance in the resurrection grasses from linking transcriptomics to metabolomics

**DOI:** 10.1186/s12870-017-1013-7

**Published:** 2017-03-28

**Authors:** Abou Yobi, Karen A. Schlauch, Richard L. Tillett, Won C. Yim, Catherine Espinoza, Bernard W. M. Wone, John C. Cushman, Melvin J. Oliver

**Affiliations:** 1 U.S. Department of Agriculture-Agricultural Research Service, Plant Genetic Research Unit, University of Missouri Columbia, Missouri, 65211 USA; 2 Nevada INBRE Bioinformatics Core, University of Nevada Reno, Nevada, 89557 USA; 3 Department of Biochemistry and Molecular Biology, University of Nevada Reno, Nevada, 89557 USA; 4 Division of Plant Sciences, University of Missouri Columbia, Missouri, 65211 USA; 5 Department of Biology, University of South Dakota, Vermillion, 57069 USA

**Keywords:** *Sporobolus stapfianus*, Resurrection plants, Gene expression, Dehydration stress, Rehydration, Abiotic stress

## Abstract

**Background:**

Understanding the response of resurrection angiosperms to dehydration and rehydration is critical for deciphering the mechanisms of how plants cope with the rigors of water loss from their vegetative tissues. We have focused our studies on the C_4_ resurrection grass, *Sporobolus stapfianus* Gandoger, as a member of a group of important forage grasses.

**Methods:**

We have combined non-targeted metabolomics with transcriptomics, via a NimbleGen array platform, to develop an understanding of how gene expression and metabolite profiles can be linked to generate a more detailed mechanistic appreciation of the cellular response to both desiccation and rehydration.

**Results:**

The rehydration transcriptome and metabolome are primarily geared towards the rapid return of photosynthesis, energy metabolism, protein turnover, and protein synthesis during the rehydration phase. However, there are some metabolites associated with ROS protection that remain elevated during rehydration, most notably the tocopherols. The analysis of the dehydration transcriptome reveals a strong concordance between transcript abundance and the associated metabolite abundance reported earlier, but only in responses that are directly related to cellular protection during dehydration: carbohydrate metabolism and redox homeostasis. The transcriptome response also provides strong support for the involvement of cellular protection processes as exemplified by the increases in the abundance of transcripts encoding late embryogenesis abundant (LEA) proteins, anti-oxidant enzymes, early light-induced proteins (ELIP) proteins, and cell-wall modification enzymes. There is little concordance between transcript and metabolite abundance for processes such as amino acid metabolism that do not appear to contribute directly to cellular protection, but are nonetheless important for the desiccation tolerant phenotype of *S. stapfianus*.

**Conclusions:**

The transcriptomes of both dehydration and rehydration offer insight into the complexity of the regulation of responses to these processes that involve complex signaling pathways and associated transcription factors. ABA appears to be important in the control of gene expression in both the latter stages of the dehydration and the early stages of rehydration. These findings add to the growing body of information detailing how plants tolerate and survive the severe cellular perturbations of dehydration, desiccation, and rehydration.

**Electronic supplementary material:**

The online version of this article (doi:10.1186/s12870-017-1013-7) contains supplementary material, which is available to authorized users.

## Background

*Sporobolus stapfianus* belongs to one of the largest and most ubiquitous families of angiosperms, the Poaceae, which includes some of the most important crop and forage species. Despite their global distribution, species in this family are generally sensitive to water deficit, and most individuals die when leaf water potentials fall below -4 MPa [1]. However, some species within the family (e.g., *S. stapfianus*), have evolved the ability to survive desiccation or the equilibration of the water potential in their vegetative tissues to that of the surrounding air (often below -100 MPa at Relative Humidity (RH) of 50% at 20 ^o^C). The roots and shoots of these species can, like seeds, remain in the dried state for considerable periods of time and when rehydrated can recover and continue growth: hence the name *resurrection* plants [2].

Desiccation tolerance (DT) developed early in the evolution of the land plants and is believed essential for the transition to dry land from fresh water [3, 4]. Vegetative DT was lost from the core lineage of the land plant phylogeny following the evolution of tracheophytes, but evolved in 13 lineages [5] within the angiosperms. In all cases investigated thus far, vegetative DT seems to have occurred by a change from developmental to environmental induction in the control networks associated with orthodox seed DT mechanisms [5, 6]. Similarities between aspects of dehydration-inducible gene expression profiles associated with vegetative DT in resurrection angiosperms and the gene expression patterns related to developmentally determined dehydration and onset of quiescence in orthodox seeds during maturation support this hypothesis [6] (and references therein).

Over the last few decades, much attention has been given to understanding the response of resurrection angiosperms to dehydration and rehydration in order to decipher the mechanistic aspects of vegetative DT [7]. Several resurrection angiosperms, primarily eudicots, have been investigated and a great deal of transcriptomic, proteomic, and metabolomic information has been obtained [7]. However, from both an evolutionary and a human societal perspective, as several major crops are monocots and employ C_4_ photosynthesis, understanding how resurrection monocots respond to the dehydration of their vegetative tissues is critically important.

The resurrection grass, *Sporobolus stapfianus* Gandoger, a member of a group of forage grasses [8] has long served as the monocot model resurrection species [9]. *S. stapfianus* has both younger desiccation tolerant (DT) and older desiccation sensitive (DS) leaves that grow on the same plant [10]. The younger DT leaves are DS if excised from the parent plant before dehydration [9]. *S. stapfianus* is easily propagated either via seed or clonally via tillers. *S. stapfianus* is also a sister species to several grasses [11] that are DS, including *S. pyramidalis* (*S. indicus var. pyramidalis*), which allows for a sister-group contrast that highlights evolutionary recent changes in function. Such a sister-group contrast strategy was used successfully to investigate adaptive metabolite signatures and phenotypes associated with dehydration by comparing the two species as they dehydrated to 60% RWC [12]. The sister-group contrast revealed some important aspects of the metabolic preparation for dehydration and the ability to respond during the initial phases of water loss that are important for the DT phenotype of *S. stapfianus*. However, it is the response to dehydration beyond 60% RWC that revealed the metabolic aspects of DT *per se*.

As *S. stapfianus* desiccates to less than 20% RWC, a complex metabolic re-programing that leads to DT is initiated and established [12]. This response is comprised of cellular protection components coupled with remobilization and retention of important nutrients, particularly nitrogen from senescing older leaves that are sensitive to desiccation. The metabolic regulation that occurs during desiccation also involved a significant investment in protection from oxidative stress (ROS) via glutathione, lipid-soluble antioxidants, and perhaps through the accumulation of gamma-glutamyl dipeptides. DT also required a large investment of carbon in the form of soluble sugars to protect cellular integrity and infrastructure as the cells dry.

In the present study, we extended our investigations into the complexity of DT by integrating a leaf transcriptomic analysis with both new metabolomics data assessing the metabolic aspects of the initiation of leaf metabolism during rehydration coupled with our previous metabolic assessments of leaf metabolism during desiccation [12]. The aim was to develop an understanding of how gene expression and metabolite profiles can be linked to generate a more detailed mechanistic model of the ways plant cells respond to dehydration, prepare for desiccation, and recover when rehydrated. To achieve this aim we combined a NimbleGen™ array approach with global metabolite profiling technologies to detail the response of the young DT leaves of *S. stapfianus* to a drying-rehydration event.

## Results

### The leaf rehydration metabolome

The results of global unbiased metabolic profiling of rehydrating dried DT young leaves of *S. stapfianus*, derived from the identical samples reported for the dehydration metabolome [12], are presented in Additional file 1: Table S1. In total, 196 different metabolites of known chemical structures were identified in the young leaf tissues, 152 of which exhibited a statistically significant alteration (*p* < 0.05 – referred to as significant in the remainder of the narrative) in abundance in one or more of the dehydration and/or rehydration treatments. The metabolite profiles of the young leaves during the dehydration process is represented as the ratio of the abundance of each metabolite in the dried state to its abundance in the hydrated state (e.g., column I, Additional file 1: Table S1 and as reported in Oliver et al., 2011 [12]. The response of the young *S. stapfianus* leaves to desiccation led to the accumulation of 50% (98 of the 196) of identified metabolites as plants approached the dried state. The rehydration metabolome was assessed by comparing the abundance of individual metabolites at 12 h and 24 h of rehydration to their abundances in both the desiccated and hydrated (pre-desiccation) states (e.g., column N, Additional file 1: Table S1). In general, the trends of the changes in abundance of many metabolites reflected the return to fully hydrated metabolic state. This included metabolites that exhibited elevated abundance during desiccation and reduced abundance relative to the dried state and continued to decline to normal levels during extended rehydration. Other metabolites decreased in abundance during desiccation and increased in abundance towards normal levels upon rehydration. However, 79 metabolites did not follow these general trends: either their abundance remained elevated or increased further during rehydration (40 metabolites p < 0.05), or became depleted or remained depleted (39 metabolites p < 0.05) during rehydration. The changes in abundance of these 72 metabolites likely highlighted processes important to the rehydration response of recovering leaf tissues (as summarized in Table 1).

**Table 1:** Metabolic response of young leaf tissues of *S. stapfianus* during rehydration from the desiccated state. Values are fold change (as a log2 value) in metabolite abundance between desiccated (DRY) and initial hydration (HYD) or between rehydration times of 12 h (R12) or 24 h (R24) and either dry or initial hydration

Super Pathway	Compound	DRY/HYD	R12/DRY	R24/DRY	R12/HYD	R24/HY
Amino acid	aspartate	**1.99**	**1.67**	**2.21**	**3.34**	**4.41**
	glutamate	**1.46**		**1.65**	**1.56**	**2.4**
	cysteine-glutathione disulfide	**1.98**	**1.62**	**2.36**	**3.21**	**4.68**
	betaine		*0.47*	*0.67*	*0.59*	
	urocanate		**4.77**	**3.77**	**4.77**	**3.77**
	phenylalanine	**3.3**	**1.22**	**2.2**	**4.04**	**7.25**
	ornithine		**2.03**	**1.89**	**2.49**	**2.31**
	stachydrine		*0.19*	*0.17*	*0.56*	*0.51*
Carbohydrate	N-acetylglucosamine		*0.28*	*0.41*	*0.36*	*0.51*
	fructose		*0.66*		*0.46*	
	fructose-6-phosphate		**3.1**	**2.6**	**3.1**	**2.6**
	glucose		*0.58*		*0.55*	
	glucose-6-phosphate (G6P)	**2.09**	**3.42**	**2.47**	**7.14**	**5.16**
	glycerate		*0.4*	*0.5*	*0.29*	*0.36*
	lactate		**2.75**	**2.23**	**3.17**	**2.57**
	pyruvate				*0.61*	*0.61*
	ribitol (adonitol)		*0.45*		*0.42*	*0.49*
Cofactors and vitamins	nicotinamide riboside*	*0.41*	*0.49*		*0.2*	*0.22*
	delta-tocopherol	**89.72**	**2.1**		**188.14**	**145.6**
Energy	mesaconate (methylfumarate)		*0.5*	*0.41*	*0.64*	*0.52*
	phosphate		*0.04*	*0.1*	*0.05*	*0.13*
Lipid	malonate (propanedioate)	**1.51**	*0.41*	*0.42*	*0.62*	*0.64*
	linoleate (18:2n6)	*0.52*	*0.57*	*0.59*	*0.3*	*0.3*
	palmitate (16:0)	*0.67*	*0.66*	*0.63*	*0.44*	*0.42*
	2-hyd roxy pal m itate	**1.45**	*0.23*	*0.4*	*0.33*	
	margarate (17:0)		*0.55*	*0.49*	*0.42*	*0.37*
	choline phosphate		**1.92**	**4.41**	**2.14**	**4.92**
	glycerol		*0.59*		*0.51*	
	glycerophosphorylcholine (GPC)	*0.21*	*0.25*	*0.39*	*0.05*	*0.08*
	1-stearoylglycerol (1-monostearin)		**2.51**		**4.29**	
Nucleotide	2’-deoxyadenosine		*0.42*	*0.37*	*0.39*	*0.35*
	adenosine		*0.24*	*0.18*	*0.48*	*0.36*
	2’-deoxyguanosine		*0.62*	*0.6*	*0.48*	*0.46*
	guanosine	*0.7*	*0.53*		*0.37*	*0.63*
	guanosine-S'^'-cyclic monophosphate (cGMP)		*0.24*	*0.26*	*0.24*	*0.26*
	allantoin		*0.11*	*0.3*	*0.16*	
	2’-deoxycytidine	*0.36*	*0.47*	*0.41*	*0.17*	*0.15*
	cytidine		*0.49*	*0.51*	*0.44*	*0.46*
	thymidine		*0.6*		*0.43*	*0.46*
Xenobiotics	glycerol 2-phosphate		*0.32*	*0.32*	*0.2*	*0.2*

Cells with values in bold indicate statistically higher levels of a metabolite (*p* <0.05) in rehydrating leaf tissue compared to either the level of the metabolite in desiccated leaf tissue or the hydrated control leaf tissue. Cells with values in italics indicate lower levels of a metabolite with (*p* <0.05) in rehydrating leaf tissue compared to either the level of the metabolite in desiccated leaf tissue or the hydrated control leaf tissue. In the DRY/HYD column the red and green shading indicates significantly (*p* < 0.05) higher and lower levels respectively of a metabolite in the desiccated leaf tissue compared to the hydrated control leaf tissue. Cells without values do not indicate a lack of measurement but rather they indicate that the difference in abundance between the two tissues in the comparison is not statistically significant (*p* > 0.05)

Eleven of the 40 metabolites that were elevated during rehydration actually increased in abundance as rehydration progressed, while the remaining 29 remained at the same elevated level reached during dehydration and did not return to normal levels. Of these 11 elevated metabolites five were amino acids or their derivatives (phenylalanine, aspartate, ornithine, trans-urocanate, and cysteine-glutathione disulfide), three were carbohydrates (fructose-6-phosphate, glucose-6-phosphate, and lactate), two were lipids (choline phosphate and 1-stearoylglycerol), and one was classified in the cofactors, prosthetic groups, electron carriers category (delta tocopherol). The majority of the 20 metabolites that remained elevated during the rehydration period also represented these four major groups: amino acids and derivatives, carbohydrates, lipids, and cofactors, prosthetic groups, electron carriers.

Rehydration resulted in the reduced relative abundance of 27 metabolites (*p* < 0.05) and the maintenance of levels already reduced as a result of desiccation of 12 other metabolites during the first 24 h of the rehydration treatment. The majority of the metabolites that decline or continued to decline upon rehydration belonged to either nucleotide or lipid metabolism pathways. Metabolites associated with purine and pyrimidine metabolism included 2’-deoxyadenosine, 2’-deoxyguanosine, adenosine, adenosine-2’,3’-cyclic monophosphate, allantoin, guanosine-3’,5'-cyclic monophosphate (cGMP), 2’-deoxycytidine, cytidine, cytidine 2’,3’-cyclic monophosphate, and thymidine. Those involved in lipid metabolism included malonate, 2-hydroxypalmitate, margarate, palmitate, glycerol 2-phosphate, glycerol 3-phosphate, glycerophosphorylcholine (GPC), and two products of lipid peroxidation 9-hydroxy-10, 12-octadecadienoic acid (9-HODE) and 13-hydroxy-9,11-octadecadienoic acid (13-HODE). Several lipids (e.g., 8-hydroxyoctanoate, linoleate, linolenate, and glycerophosphoglycerol) also remained low as compared to their levels in the desiccated state and hydrated controls.

Other metabolites that remained depleted upon rehydration included dehydroascorbate, N6-acetyllysine, glutamine-leucine, stachydrine, 5-methyl-2’-deoxycytidine, riboflavin (vitamin B2), and oxaloacetate. Notably, inorganic phosphate levels decreased significantly during the first 24 h of rehydration.

### The transcriptome of dehydration and rehydration

RNA samples extracted from hydrated, dehydrated, desiccated, or rehydrated leaf tissues of *S. stapfianus* were pooled and used to generate a catalog of 843,778 ESTs by 454 parallel sequencing (Table 2). After removal of low-quality reads, 693,236 (82.2%) ESTs were retained of which 490,144 could be assembled into 50,690 contigs. Assembled reads ranged from single reads of 99 bp to contigs up to 4,832 bp in length, with an overall average contig length of 384 bp. Sequence analysis using BLASTX against the NCBI non-redundant protein database showed that out of the 50,690 assembled contigs, 22,339 (44%) contigs predicted protein sequences that bore homology to one or more known proteins. The majority of these contigs, 16,730 (74.9%), showed predicted protein sequence similarity to those encoded by known plant genes and all matched sequences within the grass databases. The order of contig representation frequency was *Setaria italica* > *Oryza sativa* > *Sorghum bicolor* > *Zea mays* as the order of contig representation frequency. Gene Ontology (GO) classification terms could be reliably assigned to 10,143 of the 22,339 ESTs that generated positively identified in BLASTX analysis. GO term enrichment analysis (Additional file 2: Table S2) indicated that the EST collection, as a representative sample of the transcriptome, broadly represented all of the major GO classification categories. Comparison of conceptual translations to entries in the TIGRFAM, SUPERFAM, PFAM, and PRINTS databases by InterProScan identified 6,758 contigs that matched one or more domain or motif signatures (Additional file 3: Table S3). Within this group, 917 contigs had one or more pathway/reaction components that could be mapped to one or more pathway or reaction components at KEGG, UniPathway, MetaCyc, or Reactome categories (Additional file 3: Table S3). This analysis revealed a broad representation of pathways including purine and pyridine metabolism (DNA-directed DNA polymerase), photosynthesis-related pathways (photosystem and carbon fixation), carbohydrate metabolism, membrane transport pathways, energy metabolism, redox homeostasis, and amino acid or protein synthetic pathways.

**Table 2:** A summary of the 454 Life Science (Roche) sequencing analysis of the cDNA derived from pooled RNA isolated from leaf tissues subjected to different levels of dehydration and rehydration of young leaves of *S. stapfianus*

	Ss 454 GS-FLX 1	Ss 454 GS-FLX 2	Combined
Number of raw reads	435,320	408,458	843,778
Number of clean reads	343,902	349,334	693,236
Percentage of clean reads	79	85.5	82.2
Mean length of clean reads	211	212	211-212
Median length of clean reads	219	222	219-222
Total nucleotides of clean reads	72,585,449	74,102,590	146,688,039
Contigs	29,237	21,453	50,690
Reads used to generate contigs	251,297	238,847	490,144
Total contig length	11,050,617	8,422,243	19,472,860

### Identification of transcripts that increase or decline in abundance in response to dehydration or rehydration of young *S. stapfianus* leaves

**Fig. 1:**
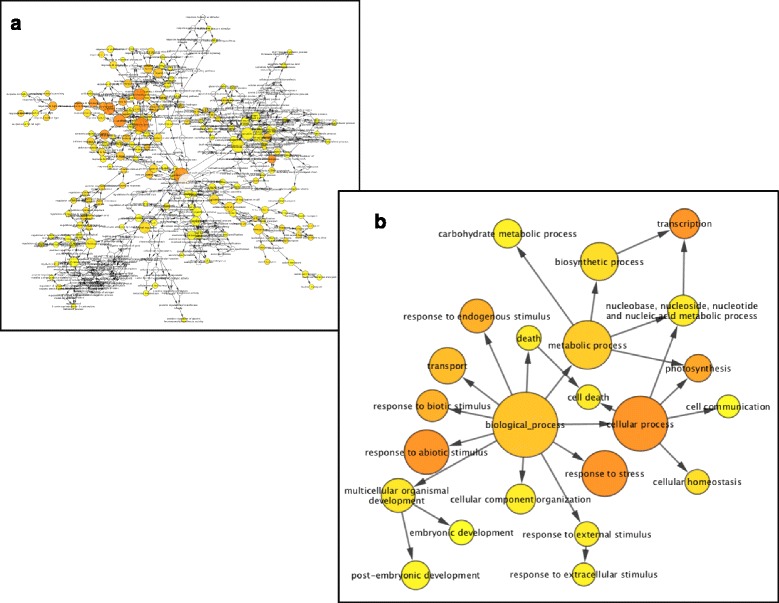
Gene Ontology (GO) terms associated with significantly differentially abundant transcripts (SDATs) surveyed during the dehydration of young leaf tissues of *S. stapfianus*. Biological Network Gene Ontology (BiNGO) was used to determine biological process terms in the full GO terms (**a**) or the GO Slim plant terms (**b**) that were enriched (*p* < 0.05). Node size represents the number of genes within the node and the color filling represents the *p*-value (at *p* < 0.05), the darker the shade the lower the *p* value

The transcript expression profiles of hydrated unstressed young DT leaves of *S. stapfianus* (at full hydration (HYD) at 96% relative water contents (RWC) were compared with those of young leaves from plants dehydrated to 80%, 60%, 40%, 30% RWC, and desiccated (DRY) at 11%RWC and at two time points after rehydration of dried plants (12 and 24 h). All quoted RWC values vary between +/- 2%. All plants were grown under greenhouse conditions and harvested at the same time of day to ensure that natural circadian rhythms in transcript abundance were not confounders in the analyses. After quality control and normalization, log-transformed expression values were subjected to an ANOVA to determine those transcripts, represented by the corresponding probe sets, that were differentially expressed across the various levels of hydration and times following rehydration. A False Discovery Rate (FDR) correction for multiple-comparisons was applied to the p-values of the ANOVA to reduce the likelihood of false positives. Transcripts were designated as statistically *significantly differentially abundant transcripts* (SDATs) if they exhibited a statistically significant change in transcript abundance during either dehydration or rehydration. A total of 4,739 transcripts were designated as SDATs, each exhibiting a statistically significant differential abundance in at least one of the treatments (Additional file 4: Table S4a). A Tukey’s (HSD) post-hoc test was performed on these 4,739 transcripts to determine under which comparison of specific treatments each was differentially expressed (Additional file 4: Table S4a). Of the 2,391 transcripts with annotation, all were differentially abundant in the dehydration series (Additional file 4: Table S4b), and 2121 were differentially abundant in the rehydration series (Additional file 4: Table S4c). Biological Network Gene Ontology (BiNGO) analysis of gene ontology (GO) terms of the annotated SDATs showed that contigs related to ‘response to stimulus’ were the most highly enriched followed by ‘transcription’, ‘photosynthesis’, and ‘transport’. SDATs in several subcategories of ‘response to stimulus’ were also highly enriched, including ‘response to abiotic stimulus’, ‘response to endogenous stimulus’, ‘response to chemical stimulus’, and ‘response to general stress’ (Fig. 1).

For most transcripts that significantly increased in abundance during dehydration, the peak of transcript abundance occurred when leaves reached approximately 30% RWC (Additional file 4: Table S4). This is also the water content at which transcripts that decline in response to dehydration reached maximum depletion. The 50 SDATs that exhibited the greatest positive fold change in abundance from leaves at 30% RWC are presented in Additional file 5: Table S5a. About 50% of these SDATs were annotated as LEA proteins with ELIPs, 1-cys peroxiredoxin, and aldose reductase also exhibiting predominance. Of the 50 SDATs that exhibited the most negative fold change in abundance (Additional file 5: Table S5b), 28 were annotated as phospho*enol*pyruvate carboxylase (PEPC), the major carboxylase in C_4_ photosynthesis, with the remaining transcripts encoding enzymes involved in photosynthesis (e.g., ribulose-1,5-bisphosphate carboxylase/oxygenase (Rubisco) activase) and energy metabolism (e.g., fructose 1,6-bisphosphate aldolase).

Transcript accumulation for SDATs that represent transcripts that increase in abundance in response to rehydration tended to peak in the first 12 h following the addition of water to the dried *S. stapfianus* leaves. The 50 transcripts that exhibit the greatest fold change at the 12 h rehydration time point (Additional file 5: Table S5c) were not dominated by any particular class, but did exhibit some enrichment for transcripts involved in protein synthesis (e.g., ribosomal proteins and elongation factors), membrane transport proteins (e.g., carbohydrate transporter/sugar porter), and membrane ATPases. The SDATs that exhibited the greatest reduction in abundance were maximally depleted within the first 12 h of rehydration (Additional file 5: Table S5d) and their profiles mirrored those that were most abundant in the 30% RWC leaf samples, which were dominated by LEA, ELIP, and 1-cys peroxiredoxin transcripts.

### Functional group analysis of differentially expressed transcripts

We concentrated on a number of specific functional groups associated with dehydration and desiccation tolerance to gain a better understanding of the complex transcriptome responses of *S. stapfianus* leaves to dehydration and rehydration. We looked for linkages to cellular protection strategies and alterations in the metabolic state alterations observed previously during dehydration [12] or rehydration (see above).

### Antioxidants and their related enzymes

**Fig. 2:**
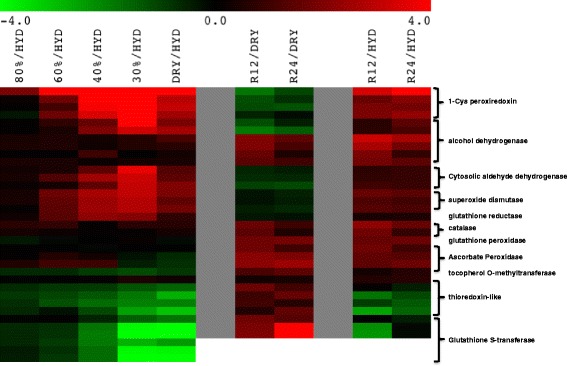
A heat map of SDATs encoding REDOX homeostasis related transcripts young leaf tissues of *S. stapfianus*. Multi Experiment Viewer (MeV 4.8.1) was used to generate the clustering of the data based on fold change values (log2). The columns represents the ratio between dehydrated (80%, 60, 40, and 30% RWC) as well as the dry state [DRY] and the hydrated state [HYD] for the first 5 columns and between either 12 and 24 h rehydration and the dry state [DRY] or initial hydrated states [HYD]. Red shading indicates a positive value for the fold change and green shading indicates negative values for the fold change in transcript abundance. Black indicates no change in transcript abundance

The majority of the SDATs that represent transcripts of genes involved in antioxidant biosynthesis pathways, reactive oxygen species (ROS) protection pathways, and redox homeostasis increased in abundance during dehydration (Additional file 6: Table S6a). The transcripts that exhibited the highest positive fold change in abundance were the 1-cys peroxiredoxin contigs (6777, 29715, 36698, 36700)), which were significantly elevated in the early phases of dehydration (80% RWC) and reached a 6.0-fold increase at 30% RWC (Fig. 2 and Additional file 6: Table S6a). The abundances of several superoxide dismutase (SODs) transcripts also increased significantly at early stages of dehydration and peaked in abundance at 30% RWC along with transcripts for alcohol dehydrogenase (contigs 9827 and 23145), cytosolic aldehyde dehydrogenase (contigs 17275, 48482, and 5987), and chloroplastic glutathione reductase (contig 47327) transcripts. Abundances of all of these transcripts decreased during rehydration, but did not fall to the levels seen in the hydrated controls. Several transcripts varied in transcript abundance to only a limited extent during drying, but were substantially elevated during rehydration. These included transcripts encoding catalase (contigs 5202 and 8896), alcohol dehydrogenase (contigs 6338, 12608, 20536, and 34062), phospholipid hydroperoxide glutathione peroxidase (contig 30123), redoxin (contig 26622), chloroplastic thioredoxin-like 3-1 protein (contig 4560), APx1-cytosolic ascorbate peroxidase (contigs 4328 and 4329), and L-ascorbate peroxidase (contig 44869).

Several transcripts encoding glutathione S-transferase, an ascorbate peroxidase (contig 12681), chloroplastic thioredoxin (contigs 14522, 23130, 23751, and 47409), and a tocopherol O-methyltransferase (contig 11580) were significantly depleted during dehydration with the maximal depletion occurring at 30% RWC, or in the desiccated state. All of these transcripts accumulated during rehydration, but did not reach the levels seen in hydrated controls, with the exception of the tocopherol O-methyltransferase.

### Carbohydrates and energy metabolism

**Fig. 3:**
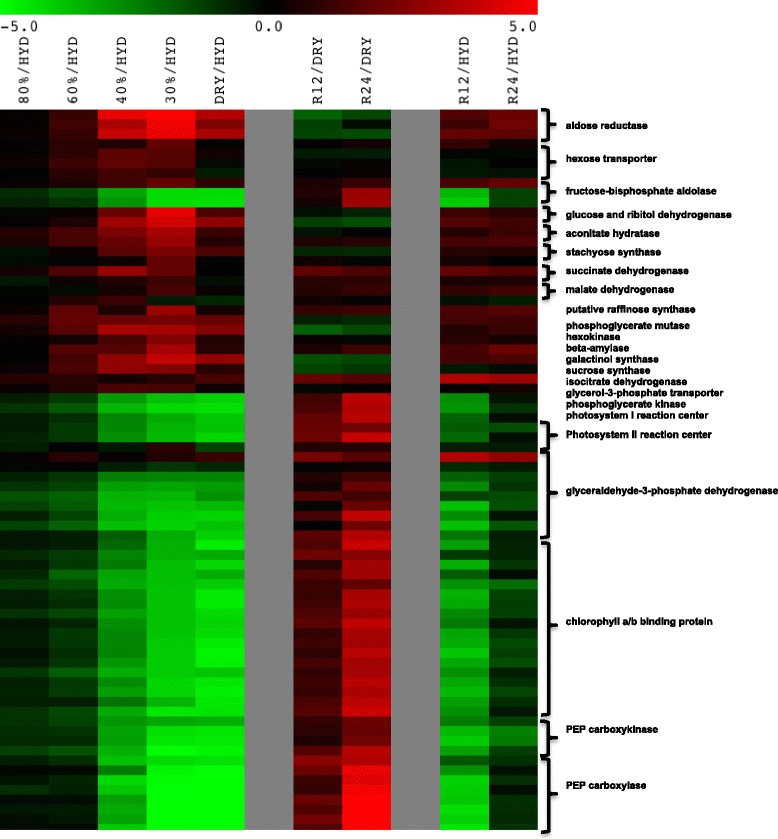
A heat map of SDATs encoding energy metabolism related transcripts young leaf tissues of *S. stapfianus*. Multi Experiment Viewer (MeV 4.8.1) was used to generate the clustering of the data based on fold change values (log2). The color code represents the ratio between dehydrated (80%, 60, 40, and 30% RWC) as well as the dry state [DRY] and the hydrated state [HYD] for the first 5 columns and between either 12 and 24 h rehydration and the dry state [DRY] or initial hydrated states [HYD]. Red shading indicates a positive value for the fold change and green shading indicates negative values for the fold change in transcript abundance. Black indicates no change in transcript abundance

SDATs associated with carbohydrate metabolism, including those involved directly in energy production and photosynthesis comprised the largest of all functional groups that responded during the dehydration of *Sporobolus* leaf tissues (Additional file 6: Table S6b). Some SDATs responding positively by an increase in abundance during dehydration encoded enzymes generally associated with carbohydrates involved in osmoprotection and glass formation during dehydration (Fig. 3). The SDATs, which exhibited the greatest fold-increase, were those annotated as aldose reductase (contigs 352, 8720, and 8722), galactinol synthase (contig 5434), and glucose and ribitol dehydrogenase (contigs 4025 and 15910) and were between 3- and 5-fold more abundant at 30% RWC than those seen in the hydrated controls. SDATs encoding beta-amylase (contig 36016), sucrose synthase (contig 6919), galactinol synthase (contig 5434), raffinose synthase (contigs 36613 and 43126), and stachyose synthase (contigs 1918 and 37122), which are associated with the synthesis of the raffinose-family oligosaccharides (RFO), as well as sucrose, also showed peak abundance at 30% RWC. Along with SDATs associated with the synthesis of important carbohydrates involved in dehydration tolerance, transcripts for hexose transporters (contigs 864, 958, 25729, and 40106), a glycerol-3-phosphate permease/transporter (contig 20058), and a sugar transporter type 2A (contig 27878) were also significantly elevated during dehydration. All of the SDATs that exhibited dehydration induced significant increases in abundance in this category decreased in abundance during rehydration, but were still more abundant at 24 h than in the hydrated controls.

Several SDATs encoding glycolytic and the TCA cycle enzymes significantly increased in abundance during dehydration. Transcripts encoding the glycolytic enzymes transketolase (contig 2008), 6-phosphofructokinase (contigs 3020 and 23024), phosphoglycerate mutase (contigs 24028 and 24443), hexokinase (contig 27198), and fructose-bisphosphate aldolase (contig 44067) all increased in abundance as dehydration reached 60% RWC and peaked at 30% RWC. Of the several SDATs that were annotated as glyceraldehyde-3-phosphate dehydrogenase only one, contig 15930, exhibited an elevation in abundance at 30% RWC. In all other samples, this SDAT was depleted in comparison to the hydrated control. In contrast, the eight other SDATs that annotated as glyceraldehyde-3-phosphate dehydrogenase were substantially depleted during all phases of dehydration. The SDATs encoding glycolytic enzymes that exhibited an increase in abundance during dehydration decreased upon rehydration, but had still not attained control levels of abundance at 24 h. SDATs that encode enzymes of the TCA cycle (e.g., malate dehydrogenase (contigs 13020 and 23772), succinate dehydrogenase (contigs 5834 and 17169), isocitrate dehydrogenase (contig 33770), and aconitate hydratase (contigs 4438 and 23348) followed a similar pattern of change in abundance during dehydration and rehydration.

SDATs encoding proteins associated with photosynthesis and carbon fixation were almost exclusively depleted during dehydration. Depletion initiated when the plants had reached 80% RWC, intensified as dehydration reached between 40% and 30% RWC, and reached a maximum depletion as desiccation ensued (DRY) (Fig. 3). Twenty-nine SDATs annotated as PEPC registered significant decreases in transcript abundance (3.8 to 6.5 log_2_ fold change) and were the most strongly affected of the 100 SDATs that exhibited the greatest decrease in abundance during dehydration. A similar number of SDATs annotated as chlorophyll a/b-binding protein transcripts were also among the most strongly affected of this aforementioned group of 100 SDATs (-3.1 to -4.8 log_2_ fold change). Transcripts encoding carbonic anhydrase (contigs 16256, 30007, and 46766), fructose 1,6-bisphosphate aldolase (contigs 6020, 31620, and 38613), fructose 1,6-bisphosphatase (contig 39525), and the chloroplastic form of phosphoglycerate kinase (contig 37468) along with transcripts encoding protein components of photosystems I and II, such as photosystem I reaction center proteins (contigs 672, 2239, 4893, and 50441), photosystem II reaction center proteins (contigs 3736, 6471, 30488, and 38381) and ferredoxin (contig 1944) all significantly declined in abundance during dehydration.

The abundances of all of the SDATs in this category that had decreased during dehydration increased during rehydration, but after 24 h were still at lower abundance than in the hydrated controls.

### Cell wall metabolism

A total of 58 SDATs were annotated as encoding enzymes involved in cell wall-associated metabolism (Additional file 6: Table S6c). Transcripts encoding enzymes involved in cell wall remodeling were among the SDATs that increased in abundance during dehydration and peaked in abundance late in dehydration when the leaves reached 30% RWC or were desiccated (DRY). This group of SDATs represented genes encoding the cell wall loosening enzyme endo-beta-mannanase (contigs 27195, 30274, 30275, and 37321) and the hydrolase beta-mannan endohydrolase (contigs 7852, 31801, and 47424). Also in this group were transcripts encoding beta-D-glucan exohydrolase (contigs 6927 and 20824), glucan endo-1,3-beta-glucosidase (contigs 12562 and 30462), feruloyl esterase (contig 14352), and glycosyltransferases (contigs 34776 and 48299). One set of SDATs encoding cell wall-associated hydrolases was clearly associated with the very early stages of dehydration and only showed increased abundance in the 80% RWC samples (contigs 2790, 7699, 16411, and 45076). At sampling times representing more advanced dehydration, these transcripts either decreased slightly or remained close to the levels in the hydrated control. Upon rehydration, the abundances of most of the transcripts that had accumulated during dehydration, including those encoding cell wall-associated hydrolases, increased further and remained at levels above those of the hydrated controls.

Ten SDATs, which decreased in abundance during leaf dehydration, were annotated as cellulose synthase, a cell wall biosynthesis enzyme (contigs 12296, 20312, 27199, 28086, 32635, 34405, 37579, 41659, 44598, and 44599). Other prominent SDATs that exhibited significant decreases in abundance during dehydration included lichenase (contigs 3275, 4266, 18174, 27060, and 30080), glucan endo-1,3-beta-glucosidase (contigs 3194, 8971, and 50667), and anthocyanidin 5,3-O-glucosyltransferase (contigs 39088 and 41715).

The majority of transcripts that were depleted during dehydration increased in transcript abundance upon rehydration, but did not reach the hydrated control levels within 24 h.

### Signaling-associated transcripts

A total of 91 SDATs encoding proteins associated with signaling kinase/phosphatase cascades, and of these, 46 increased and 45 decreased in abundance during dehydration (Additional file 6: Table S6d). Several of the SDATS that exhibited the greatest increase in abundance during dehydration started to accumulate early in the dehydration treatment at 80% RWC and peaked when dehydration reached 30% RWC. Among the SDATs that showed the greatest change in abundance were eight protein phosphatase 2C proteins (see below), an abscisic acid-inducible protein kinase-like isoform 2 (contig 7623), salt-inducible protein kinases (contigs 7610, 23041, and 23042), SNF1-related protein kinase regulatory subunit gamma-1-like proteins (contigs 3449, 30431, 39826, and 40902), a calcineurin B-like (CBL)-interacting serine/threonine-protein kinase 25 (contig 6263), MAP kinase 6 (contig 34261), a MAP kinase kinases (contigs 17651, and 28278), and an At5g01020-like serine/threonine-protein kinase (contig 39332). Several transcripts in this category, which accumulated later in the dehydration treatment at 60% RWC included two aarF domain-containing protein kinases (contigs 25016 and 25262) and a casein kinase (contig 17745), were then depleted as the leaves desiccated. Three CBL-interacting protein kinase 25 transcripts (contigs 963, 10453, and 31410), a CIPK-like protein 1 transcript (contig 37582), and a MAP kinase 5 transcript (contig 16095) all significantly increased in abundance only in the 30% RWC sample.

Some contigs represented both SDATs that increased in abundance and those that decreased in abundance during dehydration. Presumably, these SDATs are either derived from specific members of individual gene families or different spicing events. These SDATs included those encoding protein phosphatase 2C proteins: The contigs 620, 6824, 6822, 16344, 22833, 30465, 39643, 39917, and 41281 showed increased abundance during dehydration and contigs 3377, 10003, 16065, 16066, 17304, 23695, and 33258 showed decreased abundance during dehydration. SDATs encoding CBL-interacting serine/threonine-protein kinase 11 (contig 49739 accumulating and contig 18186 declining) also decreased in abundance during dehydration.

In addition to those mentioned previously, the signaling-associated transcripts that decreased in response to dehydration encoded predominantly receptor-like protein kinases (contigs 24349, 29831, 37614, and 44828), various serine-threonine protein kinases (contigs 3342, 4922, 38743, and 44752), and several leucine-rich repeat (LRR) receptor-like kinases (contigs 2513, 3324, 11992, and 17391). The transcript abundances of two CBL-interacting protein kinases (contigs 5596 and 13762), two glycogen synthase kinases (contigs 23447 and 49432), a MAP kinase 4 protein (contig 20829), and a protein kinase superfamily protein isoform 1 (contig 46776), significantly decreased under dehydration, and exhibited a small but significant increase in abundance at 60% RWC (above control levels) before decreasing below control levels as dehydration progressed.

The majority of SDATs that encoded proteins associated with signaling kinase/phosphatase cascades responded to rehydration with either increased or decreased abundance to generate a return to the levels observed in control leaves. However, there were several transcripts that increased in abundance during rehydration in continuation of a dehydration-induced increase in abundance. Two MPA kinase kinases (contigs 17651 and 37616) and a MAP kinase 6 (contig 34261), were among the most rehydration-responsive SDATs, and were 3- to 4-fold more highly expressed in rehydrated than in control samples and 2- to 3-fold more highly expressed than in the desiccated samples. Other transcripts that increased in abundance in response to rehydration included an abscisic acid-inducible protein kinase-like isoform 2 (contig 7623), casein kinase (contig 17745), CBL-interacting protein kinase 25 (contigs 6263, 31313 and 47157), CBL-interacting serine/threonine-protein kinase 11 (contig 49739), CIPK-like protein 1 (contig 37582), kinase-like protein (contig 40229), Kelch repeat: Kelch protein kinase (contig 4141), protein phosphatase 2C (contigs 16344 and 41281), serine/threonine-protein kinase SAPK6 (contig 23631), and a two-component sensor histidine kinase (contig 29491).

SDATs representing the signal transduction enzyme phospholipase D (e.g., contigs 37720, 14783, 23951, 25306, 37153, and 44461), accumulated early in the dehydration process (from 80% to 60% RWC) and peaked in abundance between 40% and 30% RWC.

### Transcription factors

**Fig. 4:**
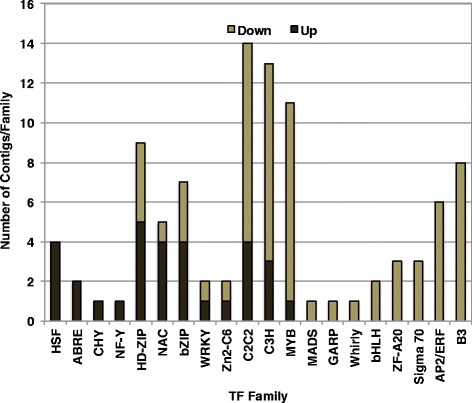
Categorization of SDATs encoding members of transcription factor transcripts representing individual transcription factor families based on their response to dehydration in young leaf tissues of *S. stapfianus*. Dark and light gray shading indicate the counts of TFs per families that increased or decreased in abundance during dehydration, respectively

A total of 102 SDATs were annotated as transcription factors (TFs), 68% of which represented transcripts that significantly decreased in abundance during dehydration (Additional file 6: Table S6e). The TF SDATs represent 20 TF families and six unclassified zinc finger proteins. SDATs representing four families of TFs (HSF, ABRE, NF-Y and CHY) all increased in abundance during dehydration, whereas SDATs representing eight other families of TFs (MADS, GARP, Whirly, bHLH, ZF-A20, Sigma 70, AP2/ERF, and B3) all decreased in abundance during dehydration. SDATs for another eight families of TFs (NAC, HD-ZIP, bZIP, C3H, MYB, C2C2, WRKY, and Zn2-C6), and the group of five SDATS encoding unclassified zinc finger proteins both increased and decreased in abundance during dehydration (Fig. 4).

Circadian clock-associated proteins (contigs 8949, 10503, and 16438), CONSTANS-like proteins (contigs 595 and 30063), and a circadian oscillator component (contig 24035) predominated among SDATs whose transcripts became most depleted during leaf dehydration. Three C3HC4 TF transcripts (contigs 7406, 28759, and 36344) were also more depleted in the dried tissues compared to control transcript abundances (-2.5 fold).

Several SDATs representing proteins involved in auxin-mediated responses decreased in abundance in all dehydrated samples and included the auxin-induced proteins (contigs 10384, 20177, and 37118), and the auxin-responsive Aux/IAA family member IAA24 (contig 14498). However, although the transcript abundances of two auxin-response factor 1 SDATs (contigs 18671 and 39205), an auxin-response factor 13 (contig 13282) and an auxin-induced protein (contig 19359) decreased upon dehydration, these transcripts exhibited a small but significant elevation in abundance relative to the control when dehydration reached 60% RWC, after which they decreased again to below control levels.

The majority of the TF transcripts that increased in abundance as *S. stapfianus* leaves dried accumulated substantially early in the drying series at 60% RWC and peaked in abundance at 30% RWC, prior to becoming depleted in the dried state. The abundance of a transcript encoding a zinc knuckle C3H protein (contig 373) increased substantially in abundance as leaves reached 80% RWC, and peaked at 60% RWC prior to a gradual decrease as dehydration continued. Thirteen other zinc finger proteins, including three transcripts annotated as CONSTANS-like proteins (contigs 30108, 30109, and 30110), that might represent a single transcript (sequential contigs) increased during dehydration. The transcripts for these CONSTANS-like proteins responded in an opposite manner to those for other transcripts also annotated as CONSTANS-like proteins (contigs 595 and 30063) in that they declined in abundance.

Also prominent among the TF transcripts that increased in abundance in response to dehydration were an ABA-responsive protein (contig 13457), an ABRE-binding factor protein (contig 22227), heat shock factor proteins (contigs 14183, 13080, and 39678), an A-6a-like heat stress transcription factor (contig 661), ATHB-6 homeobox-leucine zipper proteins (contigs 1804, 1804, and 16051), and two Tubby-like F-box protein 8-like proteins (contigs 1948, and 16196) that did not start to accumulate until the leaves had dehydrated to 60% RWC.

The abundance of almost all of the 102 TFs transcripts identified as SDATs was modulated by rehydration and returned to levels observed in the hydrated controls, but in the majority of cases failed to do so within 24h, with some exceptions. The abundances of five TF transcripts that became depleted during dehydration increased significantly to levels above those of the hydrated controls during rehydration. These transcripts include a zinc finger A20 and AN1 domain-containing stress-associated protein 11 (contigs 44210 and 44211), a Whirly family TF (contig 32848), a Zn2/Cys6 DNA-binding protein (contig 10929), and an IAA24-auxin-responsive Aux/IAA family member (contig 14498). Another five TF transcripts that increased in abundance during dehydration, but became depleted as the leaves desiccated, accumulated to levels that exceeded those of the hydrated controls upon rehydration including a ring zinc finger protein-like protein (contig 3561), a GATA transcription factor 20 (contig 21808), a BTF3-like transcription factor (contig 45428), an hsf8-like heat shock factor protein (contig 14183), and a NAC-like protein (contig 7799). In addition, six other TF transcripts, although elevated in abundance during dehydration, also continued to accumulate upon and during the 24h-rehydration period. These included the zinc knuckle C3H protein (contig 373) mentioned previously, a C6 finger domain protein (contig 30493), a WRKY 6 transcription factor (contig 28031), a BTF3 transcription factor (contig 4902), a DNA-binding protein (contig 20890), a NAC-like protein (contig 7799), and a Tubby-like F-box protein 8-like protein (contig 1948).

### Late Embryogenesis Abundant (LEA) proteins

SDATs for LEA proteins were among the most responsive groups in terms of increased transcript accumulation during the response to dehydration. The 85 SDATs representing LEA protein genes showed an increase in the associated transcript abundance early during drying as the leaves approached 80% RWC, to reach 3- to 4-fold when leaves had dehydrated to 40% and 30% RWC before decreasing slightly as desiccation approached (Additional file 6: Table S6f). LEA transcripts decreased in abundance as the leaves were rehydrated, but did not fall to the levels in hydrated controls by 24h. Group 3 LEA transcripts, represented by 47 SDATs, were among the most responsive to dehydration; 16 of the most abundant (peak expression) 25 LEA transcripts in the 30% RWC samples were Group 3 LEAs. The next largest group of SDATs encoding LEAs comprised of 25 contigs representing Group 2 LEAs (dehydrins) followed by a group comprised of 11 Group 6 LEA transcripts, designated as seed maturation proteins. Three Group 1 LEAs showed some of the highest increases in abundance that began early during dehydration and reached increases of 5- to 6-fold above control levels at 30% RWC. All of the LEA transcripts that exhibited an increase in abundance during dehydration exhibited a significant and rapid decrease in abundance within the first 12 h of rehydration from that observed in dried leaves. However, none returned to the abundances seen in hydrated controls within 24 h of rehydration

### Heat-shock proteins and molecular chaperones

SDATs encoding heat shock and molecular chaperones did not generally exhibit high-magnitude changes in abundance during dehydration with one exception: the ClpD1 chloroplastic-like chaperone protein (contig 21097) which exhibited a 3.74-fold increase in abundance in leaves dehydrated to 30% RWC (Additional file 6: Table S6g). Several small molecular weight HSPs (contigs 5583, 22979, 23945, 22978, and 37232) and three 70kD HSPs (contigs 23654, 33367, and 44609) representing the plurality of SDATs that increased in abundance in this functional category showed between 1- and 3-fold increases in abundance and the abundance of most of these peaked at 30% RWC. All SDATs representing transcripts in this subset exhibit substantial transcript depletion in the transition from 30% RWC to the desiccated state. The transcript abundances for all of the SDATs in this group decreased upon rehydration relative to those in dried leaves, but were still elevated compared to hydrated controls. Transcripts encoded by contigs 9589, 13125, 26847 and 38242 exhibited substantial depletion during dehydration, and either only partially recovered during rehydration or continued to decrease to well below hydrated control transcript abundances.

### ABA- and stress-inducible proteins

The majority of SDATs that encode transcripts that increase in abundance during dehydration and representing genes that are classified as responsive to abiotic stress, did not reach a significant fold increase in abundance of greater than 1 until the leaf tissues reached between 60% RWC and 40% RWC and the expression of most peaked at 30% RWC (Additional file 6: Table S6h). Only two of the SDATs responded positively and rapidly to dehydration and attained a significant fold increase in abundance at 80% RWC: a transcript encoding a senescence-associated protein (contigs 43784) and a transcript encoding a stress-inducible membrane pore protein (contig 44021) that showed 1.59- and 1.3-fold increases in abundance respectively. The transcript abundance of the SDAT that annotated as the stress-inducible membrane pore protein continued to increase to a peak at 30% RWC, but that of the senescence associate protein decreased as dehydration intensified but remained above that of the control. The abiotic stress-related SDATs that increased most in abundance (up to 5 fold) encode ELIPs (as described earlier) or LEA proteins, as described earlier. Other transcripts that increased significantly in abundance during the dehydration process include those encoding an ABA-induced plasma membrane protein (contig 6126), desiccation-related proteins (contig 16311 and 31699), salt and cold-induced proteins (contigs 467, 4424, 7679, 16479, 22111, 22856, 22865, 25172, 29899, and 38579), LE25-like proteins (contigs 6846, 6849, and 43912), senescence-associated proteins (contigs 513, 1844, 1845, 2141, 8866, 16098, 16099, and 39413), and HVA22-like proteins (contigs 14189 and 44034). In general, all of the SDATs in this category that increased in abundance during dehydration significantly decreased during rehydration, but did not return to the hydrated control levels of abundance.

Several SDATs that were annotated within the abiotic stress-responsive category exhibited a significant decrease in transcript abundance during dehydration. These SDATs included those that encode several salt stress-responsive proteins (contigs 1608, 4424, 15765, 26812, and 44707), an HVA22-like protein (contig 25146), two 9-cis-epoxycarotenoid dioxygenase proteins (contigs 29411 and 44138), and an ABA-induced protein (contig 5558) that started to decrease in abundance early in the dehydration treatment. Transcripts for the majority of the SDATs that decreased in abundance during dehydration are steadily replenished as rehydration advances, but do not attain levels observed in the hydrated controls, with the exception of a transcript annotated as a UVB-resistance protein (contig 29188), which remained depleted even after 24 h of recovery.

Upon rehydration, the abundances of the majority of abiotic stress-related SDATs tended to return to those of the hydrated control. However, three of the putative senescence-associated protein SDATs (contigs 6785, 13479, and 43784) and a SDAT annotated as an early drought-induced protein (contig 5517) accumulated to significant levels upon rehydration, peaking at 12 h into rehydration.

### Protein synthesis and degradation

Transcripts encoding proteins involved in protein synthesis and turnover were well represented in the dehydration and rehydration response transcriptomes (149 total) (Additional file 6: Table S6i). The majority of the SDATs (47) that were annotated as related to protein synthesis represented transcripts encoding the 40S and 60S ribosomal subunit proteins and four chloroplast 28 kDa ribonucleoproteins (contigs 6558, 18012, and 36965). The abundances of transcripts for these ribosomal proteins, in general, increased during the early stages of dehydration (from 80% to 40% RWC) and tended to remain elevated as desiccation was achieved. However, rehydration appeared to be the major trigger of an increase in abundance of these transcripts, some of which, such as the transcript for the 60S ribosomal protein L36 (contig 24330), increased as much as 5.0-fold. Transcripts for translation initiation (contigs 1079, 16555, 19023, 27086, and 40176) or elongation factors (contigs 13136, 13429, 13755, 33339, 46793, 48021, and 49190) had similar patterns of changes in abundance as those seen for the ribosomal protein SDATs. Only nine transcripts decreased in abundance at all stages of leaf dehydration including those encoding translation elongation factor G (contigs 23831 and 38651), elongation factor Tu (contigs 3063 and 23078), a translation factor chloroplastic-like GUF1 homolog (contig 25171), and the post-translational modification polypeptides ubiquitin (contig 1089) and polyubiquitin (contigs 2836, 2838, and 22823).

### Cluster analysis

Cluster analysis of transcript abundances during each stage of dehydration was performed to identify coordinated expression patterns and reveal any functional control of the response of *S. stapfianus* to dehydration that might be exerted by tissue water content. A cluster analysis for transcript abundances during rehydration was performed but, as there were only two rehydration treatments, 12 and 24 h, the analysis did not yield a robust analysis. As the quality-controlled normalized data were normally distributed, the ANOVA analysis and the choice of parametric similarity metric were meaningful. Clusters were defined at a threshold of 0.85; specifically, groups of SDATs with an average pairwise Pearson Correlation of 0.85 or more were deemed a cluster. The SDATs separated into 765 clusters in this way (Additional file 4: Table S4). Of these 765 clusters, 56 clusters contained 10 or more SDATs; these clusters represented 71% of the total SDATs. Remaining clusters contained only one or two SDATs (561 clusters of 713 or 15% of all SDATs).

Four clusters (D1, D2, D24, and D32) predominate among SDATs that exhibit increased transcript abundance during dehydration, accounting for 1,114 or 23.5% of all SDATs (Additional file 7: Figure S1A). Clusters D1 and D2 that contained SDATs that increased in abundance between 80 and 60% RWC or plateaued in abundance at 40% RWC (Cluster D1) or peaked in abundance at 30% RWC (Cluster D2). These two clusters contained most of the SDATs that encoded LEA or LEA-like proteins. Cluster D1 contained 54 LEA/LEA-like transcripts, including the majority of the Group 3 LEA SDATs, whereas Cluster D2 contained 25 LEA/LEA-like transcripts including all those encoding the group 4 LEA protein [LE-25 like] on the array. These two major clusters also contained a number of transcripts that encode proteins important in redox homeostasis pathways. The majority of the 1-cys peroxiredoxin-encoding transcripts belonged to Clusters D1 and D2 and all of the superoxide dismutase transcripts belonged to Cluster D1. Cluster D2 contained glutaredoxins, gamma-glutamylcysteine synthetase, and glutathione reductase as other representatives of redox homeostasis pathways. Cluster D2 included all but one of the SDATs encoding ELIP proteins, all of the SDATs encoding glucose and ribitol dehydrogenase, and a large percentage of SDATs encoding proteins such as aldose reductase, carboxylesterase 13-like proteins, cytochrome P450 monooxygenase, nitronate monooxygenase, peroxisomal proteins, and aldehyde dehydrogenases, which are involved in cellular detoxification pathways. Cluster D2 also contained all of the pyruvate decarboxylase SDATs and two of the alcohol deydrogenase SDATs, both of which were associated with fermentation pathways. Cluster D2 also contained several SDATS whose transcripts encoded enzymes associated with cell wall modification including all of the endo-beta-mannanase encoding SDATs, as well as those encoding glucan endo-1, 3-beta-glucosidase and glycosyl hydrolase.

Cluster D24 included SDATs that increased in abundance during the first stage of dehydration from Hydrated to 80% RWC, maintained a steady elevated transcript abundance until tissues were between 40RWC and 30% RWC followed by reduced accumulation, then by another increase in abundance as the tissues continued to dry to between 30% RWC and the desiccated state. This cluster was almost completely populated by transcripts encoding proteins involved in retrotransposon activity, gag-pol polyproteins, reverse transcriptase, and several unclassified retrotransposon proteins.

Cluster D32 included SDATs that increased steadily in abundance during the first stage of dehydration from Hydrated to 40% RWC, maintained a constant elevated abundance until tissues reached 30% RWC followed by a rapid reduction in abundance as the tissues desiccated. This Cluster contained six protein phosphatase 2C SDATs that were elevated during dehydration and three phospholipase D (50% of SDATs annotated as phospholipase D in the transcriptome) transcripts. Notably, the cluster also included several SDATs associated with carbohydrate metabolism such as sucrose synthase, hexokinase, and sugar and hexose transporters, as well as those associated with various aspects of redox homeostasis pathways such as glutathione reductase and mono-dehydroascorbate reductase. Hexose transporters and some of the transcripts associated with redox homeostasis were also featured in Cluster D31 (55 SDATs), which has a profile of transcript abundance pattern very similar to that of Cluster D32, except that transcript abundances decreased relatively rapidly between 40% and 30% RWC.

Of the clusters that that showed decreasing transcript abundance during dehydration, clusters D5 and D13 accounted for 792 SDATs 923.5% of the total) and all of the SDATs with no elevation in transcript abundance at any stage during dehydration (725) (Additional file 7: Figure S1B). Clusters D5 and D13 showed mutually similar patterns of transcript abundance during dehydration. Transcript abundances in these two clusters decreased slightly or remained constant as the plants dried from Hydrated to 60% RWC, followed by a rapid decrease in the tissues dried between 60% and dryness. The transcript abundances of SDATs within Cluster D13 decreased more precipitously than did those in D5. Cluster D5 included SDATs representing transcripts that encoded proteins directly associated with photosynthesis including 31 of the 36 SDATs encoding chlorophyll a/b binding proteins, photosystem I reaction center subunits, 24 of the 26 SDATs encoding PEPC, all of the SDATs encoding phosphoenolpyruvate carboxykinase, all SDATs encoding photosystem II reaction center W protein, all but one of the SDATs encoding carbonic anhydrase, Rubisco small subunit, and Rubisco activase (see Additional file 6: Table S6b for a full list). This cluster also contained all but one of the SDATs encoding sucrose-phosphate synthase. Cluster D13 also contains all of the SDATs encoding photosystem I reaction center subunit III and the majority of those encoding ferredoxin, but has a much more varied membership.

### Validation of expression analysis data

**Fig. 5:**
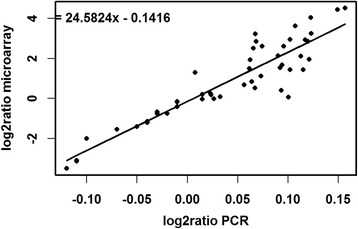
A linear regression graph between transcript abundance derived from the qRT-PCR experiment (X-axis) and transcript abundance calculated from microarray analysis (Y-axis). Here, R^2^ = 0.843, and the non-parametric Spearman correlation coefficient is 0.901. Each symbol represents the log2 ratio of each average of the conditions 80%, 60, 40, 30% RWC with respect to the average hydrated state [HYD] expression value for each of the 10 target genes described in Additional file 9: Table 7

To validate the NimbleGen array data, a qRT-PCR analysis was performed for a set of SDATs. The results presented in Fig. 5 (Additional file 8: Table S8) demonstrate a strong correlation between the transcript expression values and trends in accumulation and depletion between the two methods of analysis (goodness of fit R^2^ = 0.843 and non-parametric Spearman correlation coefficient of 0.901).

### Endogenous ABA

**Fig. 6:**
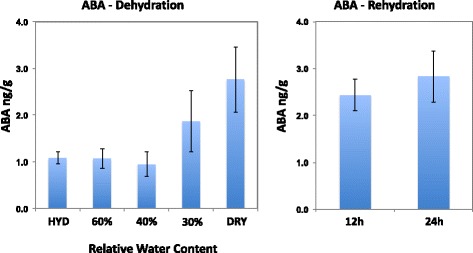
Endogenous ABA concentrations on a dry weight basis in young leaf tissues of *S. stapfianus* during dehydration (A) and rehydration (B). Analysis was conducted on hydrated tissues (HYD), tissues dehydrated to 60%, 40%, and 30% RWC as well as dry tissues [DRY]. Data are the means ± SD (*n* = 8)

Leaf endogenous ABA concentrations were determined during the drying process and upon rehydration (Fig. 6). ABA concentrations rose steadily during the drying process to reach a maximum concentration of approx. 2.5 ng/g Dwt in desiccated leaves, matching results of previously reported analyses [13]. The increased ABA concentrations were maintained throughout the 24 h rehydration period with perhaps a slight increase in the latter 12 h to approx. 2.8 ng/g Dwt.

## Discussion

The 454 sequence-generated transcriptome comprised 50,690 unique contigs that formed the basis of the NimbleGen oligonucleotide array used to measure gene expression reported here. Although the gene expression analysis array technology is currently less used, it allowed for the analysis of large numbers of samples and less of a requirement for a reference genome sequence. It is not yet possible to determine what the coverage of the gene content of the *S. stapfianus* genome represented by these contigs. Given the tetraploid genome size of 2.67 pg/2C [11], or haploid size of 1.3 Gb (data not included), it is highly unlikely that this transcriptome provides full representation. The annotation also reveals some apparent redundancy within the contig collection. However, it also clear from the functional group analyses and GO assignments of the 22,339 annotated contigs that the 454-generated transcriptome provided broad transcript representation of the functional cellular components and metabolic pathways and delivers a useful assessment of the response of *S. stapfianus* to both desiccation and rehydration. The 454-generated transcriptome described here is also similar in content and functional coverage to those reported for other resurrection angiosperms (as discussed in [7]). Given the recognized limitations of the NimbleGen array technology, as described by Mutz et al 2013 [14], this expression analysis does not provide a detailed assessment of the genetic aspects of the responses of *S. stapfianus* leaves to desiccation and rehydration, but does provide a solid platform for evaluating the functional roles that candidate genes play in the physiological and cellular perturbations during these responses in leaves.

### The dehydration and desiccation response

The metabolome of dehydrating and desiccated leaves of *S. stapfianus* was detailed in an earlier report [12] and is represented in Additional file 1: Table S1. The analysis of the metabolome during drying revealed a number of significant perturbations as water is removed from the tissues. A primary response to dehydration was a substantial and steady accumulation of several amino acids, gamma-glutamyl dipeptides, carbohydrates, and anti-oxidants that reached maximum accumulation as the tissues dry (Additional file 1: Table S1). These compounds appear critical for the desiccation tolerance phenotype of *S. stapfianus*, either directly by facilitating cellular tolerance or indirectly by providing a source of metabolites necessary for recovery and subsequent whole-plant survival [5].

Amino acids start to increase in abundance early in the dehydration process presumably to fuel osmoregulation and resist water loss [12]. However, the transcriptome does not appear to reflect this process, suggesting that this early increase in amino acid concentrations is under metabolic control and is not fueled by an increase in the appropriate transcripts. The continued accumulation of amino acids in the young leaves of *S. stapfianus* as they approach desiccation is thought to be the result of nitrogen mobilization fueled by protein degradation that occurs during dehydration [15]. Protein degradation occurs in young leaves during dehydration, although to a minor degree compared to that seen in mature desiccation-sensitive leaves that senesce as they dry [15]. This suggests that the accumulating amino acids are not primarily a result of increased biosynthesis, but rather of transport into the young leaves for storage to fuel growth when the plants are rehydrated. The transcriptome of the leaves undergoing dehydration appears to support this hypothesis as the abundance of transcripts encoding amino acid biosynthetic enzymes was generally unaltered (Additional file 1: Table S1), whereas that of transcripts encoding enzymes involved in protein degradation (Additional file 6: Table S6i) are elevated and within the collection of SDATs. Storing nitrogen in the form of amino acids in the roots and crown tissues under stress conditions is a common adaptive tool that perennial grasses use to support new growth when conditions become favorable [16]. Thus, it appears that *S. stapfianus* sequesters amino acids in the younger desiccation-tolerant leaves rather than in the roots and crown tissues as part of its strategy for surviving desiccation. *S. stapfianus*, along with other desiccation tolerant species also accumulates gamma-glutamyl dipeptides during dehydration, nitrogenous compounds involved in redox potential homeostasis in animal tissues [17], although perhaps not in plants [18]. Unfortunately, the 454-derived transcriptome that we developed for this study does include a transcript for the gamma-glutamyl transpeptidase (GGT) the enzyme responsible for their synthesis. Thus we are unable to assess the role that transcript accumulation could play in this aspect of the desiccation response in this study.

**Fig. 7:**
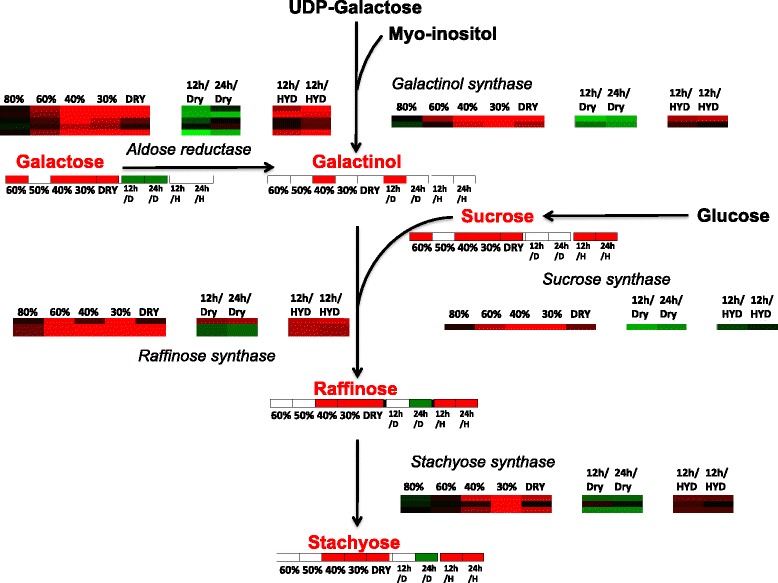
Concordance between the abundance of intermediates in the biosynthesis of Raffinose Family Oligosaccharides (RFO) and the transcripts encoding the enzymes of the pathway during the dehydration-rehydration cycle in young leaf tissues of *S. stapfianus*. A single longitudinal bar directly under the compound in the pathway represents the metabolite abundance heat map for each compound. The data used to generate the metabolite heat maps are reported in Oliver et al 2011. Red shading indicates a statistically significant fold increase in abundance and green shading a statistically significant fold decrease in metabolite abundance. White indicates no change in metabolite abundance. For the dehydrating samples (60% RWC to DRY) the fold change is given in relation to the hydrated control, where red shading indicates a statistically significant increase in abundance compared to the hydrated control for the dehydrating samples. For the rehydrating samples the fold change is given in relation to either the dry samples (/D) or the hydrated control (/H). The longitudinal bars directly associated with the enzyme identity in the pathway represent the transcript abundance heat map for each enzyme. Each line in the heat map constitutes a single contig annotated as encoding the associated enzyme. Red shading indicates a statistically significant the log_2_-fold increase in abundance and green shading a statistically significant the log_2_-fold decrease in abundance. Black indicates no change in transcript abundance. For the dehydrating samples (80% RWC to DRY) the log_2_-fold change in abundance is given in relation to the hydrated control. For the rehydrating samples log_2_-fold change in abundance is given in relation to either the dry samples (/DRY) or the hydrated control (/HYD)

The accumulation of sugars in vegetative tissues during the drying process is a common feature of desiccation-tolerant plants [7]. in *S. stapfianus,* sucrose steadily accumulates during drying, and then raffinose, stachyose, and the sugar alcohols galactinol and myo-inositol accumulate as desiccation is approached ([12]; Additional file 1: Table S1). Within the DT trait, sugars, in particular sucrose, have been associated with intercellular glass formation that is postulated to slow chemical degradation and prevent membrane collapse during dehydration [19, 20]. Raffinose is reported to enhance this function of sucrose [21] and galactinol and raffinose have been demonstrated to display anti-oxidation capabilities *in vitro* [22] as has myo-inositol [23]. Raffinose and stachyose also contribute to cellular stress protection and carbon storage [12, 24–26]. Unlike the enzymes involved in amino acid biosynthesis, the transcripts for many of the enzymes involved in sucrose biosynthesis and in the Raffinose Family Oligosaccharide (RFO) pathway accumulated during dehydration (Fig. 7). The increased abundance of transcripts encoding sucrose synthase, galactinol synthase, raffinose synthase, and stachyose synthase demonstrate the importance of RFO intermediates in the DT phenotype. Sucrose synthase and stachyose synthase also accumulate during the dehydration of *Haberlea rhodopensis* and *Craterostigma plantagineum* [27]. Transcripts encoding sucrose synthase also accumulated in the initial phases of dehydration, between hydrated and 60% RWC (Additional file 7: Figure S1A: Cluster D32), along with transcripts encoding several sugar transporters, which implies that the sucrose is likely transported both within the young leaves and to them. Whittaker et al. (2007) [28] concluded that the rapid loss of starch during dehydration of *S. stapfianus* leaves fuels the steady increase in sucrose (and perhaps also the accumulation of amino acids); however, the transcriptome analysis reported here did not present such a clear finding. Transcripts for the starch-degrading enzyme beta-amylase varied in their response during dehydration. Of the five SDATs identified as beta-amylase four represent transcripts that decreased in abundance during dehydration (Additional file 7: Figure S1B: Clusters R13) and only one represents transcripts that accumulated during drying (Additional file 7: Figure S1A: Cluster D8), peaking late in the drying process at 30% RWC. The late increase in transcript abundance for the only positive beta-amylase SDAT, the decrease in abundances of other beta-amylase transcripts, and the increase in abundance of an alpha-amylase inhibitor protein (contig 22951; Cluster 31) would suggest that the early and rapid loss of starch in the leaves of the drying plant is under metabolic control. The increased abundance of the transcript encoding hexokinase throughout the dehydration process might also have contributed to the accumulation of sucrose and subsequent RFO at the expense of reducing sugars such as glucose and fructose. The activity of this enzyme increases during the dehydration of *S. stapfianus* and *X. viscosa,* highlighting its importance in lowering the toxicity of reducing sugars in the dehydrating tissues [29].

The dehydration-induced increase in the abundance of several transcripts encoding aldose reductase, a highly dehydration-responsive SDAT (Additional file 7: Figure S1A; Clusters D1, D2, and D32), and rate-limiting enzyme in polyol biosynthesis [30] could explain the accumulation of polyols (e.g. arabitol and mannitol), generally considered as osmolytes, in the dehydrated leaves of *S. stapfianus* [12] and indicates a possible role for these compounds in the DT of this species.

The rapid catabolism of starch reserves during drying results in early accumulation of both maltose and glucose [12], which drives the flow of carbon through glycolysis and the TCA cycle and generates reducing power and ATP [31]. Presumably, this process generates the energy required to prepare cells to survive desiccation and to maintain cellular integrity in the dried state. The flow of carbon from glycolysis into the TCA cycle results in an accumulation of the TCA cycle intermediates citrate, cis-aconitate, alpha-ketoglutarate, succinate, fumarate, malate, and as well as glucose 6-phosphate (glycolysis) as the leaves approach desiccation [12]. The critical role of glycolysis and the TCA cycle during dehydration is supported by the significant changes in the transcriptome, with increases in the abundances of several transcripts encoding key enzymes in these pathways in the early stages of dehydration, and peaks in their abundances just prior to the final stages leading to desiccation.

Although the generation of reducing power and ATP during dehydration is probably the critical role for the TCA cycle in the establishment of cellular desiccation tolerance, the cycle might also direct metabolites to other important processes necessary for cellular well-being during dehydration. The TCA cycle provides carbon skeletons for nitrogen assimilation through alpha-ketoglutarate (2-oxoglutarate) and oxaloacetate (OAA) [32], which are branch points for amino acid biosynthetic pathways. Although the amino acids that accumulate in dehydrating young leaves of *S. stapfianus* could be derived from nitrogen mobilization from protein catabolism, there is also some evidence that amino acid biosynthesis also occurs during dehydration, at least in the early stages [15, 33]. Such a scenario might explain the lack of accumulation of OAA during dehydration and a twofold decline in this TCA intermediate in the dry state. The accumulation of the carboxylic acid intermediates of the TCA cycle might also indicate a need to delay the loss of turgor in the leaf cells to maintain function as the plants dry or to ensure pH homeostasis [34], which is critical in maintaining metabolism during dehydration [35]. Gamma-aminobutyrate (GABA), which accumulates during dehydration and is closely associated with the TCA cycle via the GABA shunt, also has a proposed role in maintaining cellular pH during stress [36]. There is not a great deal of evidence that the importance of these processes is reflected in the transcriptome; however, the transcript of a vacuolar proton ATPase (contig 31537) steadily accumulated during dehydration, as did that of a tonoplast dicarboxylate transporter (contig 45651), which increased early in dehydration before it began to decrease as the leaves desiccate. Transcripts for glutamate decarboxylase (contig 31842), which catalyzes the conversion of glutamate to GABA and was classified as a Cluster 2 SDAT (Additional file 7: Figure S1), accumulated during dehydration.

Metabolite analyses of dehydrating *S. stapfianus* leaves suggested that the glutathiones and tocopherols, presumably situated in the cytoplasm and membranes respectively, were important components for protecting cells against dehydration-induced accumulation of ROS [12, 37]). A role of the glutathione in protecting cells from ROS activity and maintaining redox homeostasis is a common feature of resurrection species [38] and tocopherols have long been implicated in maintaining membrane integrity under the threat of ROS during seed desiccation [39, 40]). The transcript profiles observed here appear to support the observed increases in these metabolites with significant increases in the abundance of transcripts encoding glutathione synthase (GS), γ-glutamyl cysteine synthetase (γ-GCS) and glutathione reductase (GR), and tocopherol cyclase, the enzyme that converts various phytyl quinol pathway intermediates into their corresponding tocopherols [41].

Concentrations of ascorbic acid, a key component of a redox hub responsible for integrating metabolic responses to environmental stimuli via a cellular signaling network [42], remains essentially unchanged throughout the dehydration (and rehydration) process. However, dehydration does result in a significant increase in one of the precursors of ascorbate, gulono-1,4-lactone, and a significant accumulation of threonate, a catabolite of ascorbate [43], which indicates that ascorbic acid metabolism is in flux during the dehydration-rehydration cycle and might play a role in the desiccation tolerance mechanism in *S. stapfianus*.

The transcriptome provides further evidence of a central role for redox homeostasis and protection from the damaging effects of ROS production during dehydration in the desiccation tolerance phenotype of *S. stapfianus*. The dehydration-induced disturbance of cellular homeostasis resulted in the deployment of several enzymatic antioxidants along with the aforementioned metabolites to control the damaging effects of toxic reactive oxygen intermediates [44]. In the transcriptome of the dehydrating *S. stapfianus* leaf, transcripts encoding thiol-based peroxidases, 1-cys peroxiredoxins, represented the most desiccation-responsive SDATs that accumulate during dehydration. The 1-cys peroxiredoxins reduce H_2_O_2_ and alkyl hydroperoxides [45] and appear to be dehydration-induced in both seeds and resurrection plants, but not in the vegetative tissues of DS species even under stress conditions [46, 47]), which suggests their critical role in desiccation tolerance. Several superoxide dismutase (SODs) transcripts also increased significantly in abundance during dehydration in *S. stapfianus* leaves, as was seen in dehydrating *Xerophyta viscosa* [48], which indicates a need to prevent the activity of oxygen free radical activity during dehydration in resurrection species [49]. Although not strictly an anti-oxidant, transcript abundance for the detoxification enzyme aldehyde dehydrogenase also increases significantly during desiccation (Cluster 2) and has been associated with the desiccation response in a diverse range of resurrection species, including *Craterostigma plantagineum* [50] and the moss *Syntrichia (Tortula) ruralis* [51]. This enzyme is postulated to detoxify excess aldehydes that accumulate as metabolism is disrupted during dehydration [52].

Along with the 1-cys peroxiredoxins, transcripts encoding ELIPs comprised a significant proportion of the most abundant SDATs. ELIPs are proteins that are believed to bind chlorophylls as a means to protect chloroplasts from ROS-induced photo-oxidative damage [53] and their transcripts have been reported to increase in abundance in the leaves of several resurrection species, including *Boea hygrometica* [54], *Haberlea rhodopensis* [27], and *Craterostigma plantagineum* [50]). Neale et al., 2000 [55], using a cold-plaque technique, cloned an ELIP cDNA from *S. stapfianus* that was induced in leaf tissues during dehydration. The timing of the accumulation of this ELIP transcript was important as it coincided with the beginning of the non-photochemical quenching period during dehydration [13]. The transcript encoded by the ELIP cDNA did not accumulate in response to dehydration in detached leaves of *S. stapfianus (*detached leaves do not exhibit DT), nor could stress-inducible homologous transcripts be detected in water-deficit stressed DS *Sporobolus pyramidalis*. The commonality of desiccation-induced accumulation of ELIP transcripts in resurrection plants testifies to their importance in desiccation tolerance strategies and the need to protect chloroplast membranes from ROS-induced damage [7].

Clearly, mechanisms that limit cellular damage due to ROS during dehydration of leaf tissues are an important component of desiccation tolerance in many, if not all, desiccation-tolerant plants and tissues. In leaf tissues, ROS are thought to be produced primarily through the disruption of normal electron flow through Photosystems I and II and the resulting increases in both photoreduction of oxygen and photorespiration during dehydration and declining CO_2_ assimilation [56]. In resurrection species, this process is amplified when dehydration of leaves occurs under high light conditions, which is typical of their natural habitats. To add to the likelihood of ROS production and injury, *S. stapfianus* is a homeochlorophyllous resurrection species that retains the majority (approximately 60%) of its chlorophyll [57] and preserves chloroplast integrity [58] during dehydration and in the dried state. As for all resurrection species studied so far [49], photosynthesis in *S. stapfianus* is very sensitive to dehydration and shows a rapid and linear decline in CO_2_ assimilation to complete cessation at approximately 45% RWC (~1.0 gH_2_O/g dwt) [13]. This sensitivity was manifested in the transcriptome as significant depletion of transcripts encoding many major components of the photosystems, enzymes of carbon fixation, and the Calvin cycle. Transcripts for PEPC, the key carbon fixation enzyme in C_4_ species (and that also regulates flux through the TCA cycle), were rapidly depleted during dehydration (Cluster D5) and were the most depleted of all of the dehydration-repressed SDATs. Mirroring this decline was the repression of transcripts for other proteins involved in carbon fixation (carbonic anhydrase, Rubisco small subunit, and the Rubisco activase), and photosystem structure (CAB proteins, ferredoxin, and photosystem I and II reaction center proteins), all in clusters D5 and D13. The loss of these transcripts is unlikely to be the direct cause of the cessation of carbon fixation during drying [13], but it does signify a programmed repression of gene expression associated with processes that are either untenable or harmful during dehydration and a switch of available resources to the accumulation of transcripts that are necessary for surviving the desiccated state.

*S. stapfianus* has evolved morphological strategies to limit the absorption of light energy and the likelihood of ROS generation in leaf tissues when desiccated. This species does so by retaining a large proportion of leaf chlorophyll and preserving thylakoid membranes during drying and in the dried state to enable rapid resumption of photosynthesis upon rehydration [13]. As *S. stapfianus* dries, differential contraction of rows of leaf cells cause the leaf blades to curl such that below 44% RWC leaf margins curve over one another. At even lower RWC the leaves become spike-like [9]. The result of this morphological change, in the context of light production of ROS in high light, is a significant decrease in the surface area of the leaf available for light absorbance as well as a more upright stature that decreases exposure to direct sunlight and reduces leaf temperatures. Although initially driven by differential cell contraction, the folding of cell walls and the modification of cell wall properties are thought to play a major role in dehydration-induced changes in leaf morphology [38, 59]). The metabolome of dehydrating *S. stapfianus* leaves revealed a late accumulation of ferulate and caffeate, precursors of cell wall lignin and cross-linking compounds (Additional file 1: Table S1) indicating, perhaps, an inhibition of lignin biosynthesis and a decrease in cell wall cross-linking. That the latter might be the case is suggested by the increased abundance of transcripts for feruloyl esterase (Contig 4352) during dehydration. Feruloyl esterase catalyzes the release of ferulate and ferulate dehydrodimers from cell walls of grasses by the cleavage of ester bonds that cross-link ferulic acid to polysaccharides, primarily arabinoxylans [60], which enhances cell wall extensibility [61]. The importance of cell wall loosening in the response to desiccation, and perhaps tolerance, in *S. stapfianus* leaves is also supported by the early accumulation of transcripts encoding other cell wall hydrolases, such as endo-beta-mannanase and beta-mannan endohydrolase, along with those encoding cell wall hydrolases. Equally notable was the dehydration-induced depletion of cellulose synthase (10 SDATs) which indicates a cessation of cell wall biosynthesis.

As was expected from the transcriptome-based investigations on the response of resurrection angiosperms to dehydration and desiccation [7], leaves of *S. stapfianus* accumulate transcripts that encode LEA proteins and other ABA-responsive proteins. LEA transcripts were by far the largest group of all types of transcripts that accumulated during dehydration, particularly during early dehydration, and also recorded the highest transcript abundance throughout the dehydration process. LEA proteins are believed to protect cells from desiccation-induced damage by acting as hydration buffers or by direct protection of proteins and membranes [62], [63]. Group 3 LEA transcripts predominate the dehydration transcriptome of *S. stapfianus* and prevent or reduce protein aggregation alone or in combination with disaccharides during dehydration in vitro [64, 65]. The ubiquity of increased LEA protein transcript abundance in the transcriptomes of angiosperm resurrection species during dehydration and their classification as seed maturation proteins underpins the hypothesis that resurrection plants evolved their mechanism of desiccation tolerance from a reprogramming of the developmentally controlled seed desiccation tolerance (first documented by [66]; and discussed by Oliver et al. 2005 [4]; and more recently by Farrant and Moore 2011 [6]).

Many of the abiotic stress-related transcripts that were induced during dehydration appeared around 30% RWC. This late response to dehydration included transcripts encoding several ABA-inducible proteins such as ELIPs (see earlier), protein LE25-like, HVA22-like protein, and responsive to ABA, which is consistent with the steady increase in endogenous ABA during dehydration. Transcripts encoding several heat shock proteins (HSPs), ranging from smaller (17–19 kDa) to larger (70–101 kDa) HSPs, accumulated during dehydration and many peaked later stages of dehydration at 0.75 g H_2_O/g Dwt, a common observation for both desiccating seeds and resurrection plants [7]. HSPs, in particular the smaller chaperonins and protein disaggregates, are important components for maintaining protein homeostasis [67]. The dehydration and rehydration of cells has a major impact on protein structural integrity and aggregation that clearly must be mitigated for survival.

Many aspects of the dehydration response in *S. stapfianus*, reported here and elsewhere [5], such as elevated sugar content, alterations in protein homeostasis, and changes in hormone levels are triggers for leaf senescence [68], which suggests that delay or inhibition of senescence is a critical aspect of vegetative desiccation tolerance in this grass [69]. Neither the metabolome nor the transcriptome of the young desiccation-tolerant leaves presented here support or refute this possibility. However, we did observe an accumulation of transcripts that related to senescence, although the annotation of these transcripts simply describes cDNA sequences isolated from senescing pea pods [70], with no experimental evidence that they are actually associated with any process related to leaf senescence. We also observed an accumulation of transcripts encoding UDP-glycosyltransferase (Contig 7378), in agreement with the isolation of a dehydration-induced UDP-glycosyltransferase gene from *S. stapfianus* [71] that is postulated to metabolize strigolactone (Islam et al., 2013), which in turn might inhibit leaf senescence during drying [69]. However, the increase in abundance of this transcript is relatively modest at 0.46-fold above the maximum in the hydrated control leaves (30% RWC). Recently [72]) the induction of autophagy by a dehydration-induced accumulation of the disaccharide trehalose, which prevented the onset of senescence and programmed cell death in the leaves of the resurrection grass *Tripogon loliiformis* was reported*.* Trehalose was not detected in the leaf metabolome (Additional file 1: Table S1) of *S. stapfianus* in contradiction of earlier reports [73]. Our current results are difficult to reconcile with the previous report [73]) as the methodologies for sugar analysis differed considerably between the two studies. It is extremely unlikely that trehalose was simply undetected in *S. stapfianus* using the Metabolon mass spectrometry-based metabolomics platform (as described in the Materials and Methods), as it is identical to that used for analysis of samples from the resurrection lycophyte *Selaginella lepidophylla*, in which trehalose was evident [74]. We have also identified trehalose in root samples of *S. stapfianus* using the Metabolon platform (Yobi et al., unpublished data). Thus, if autophagy is induced in *S. stapfianus* leaves during dehydration to inhibit dehydration-induced senescence, and trehalose is absent in the leaves of *S. stapfianus* during dehydration, then a different signaling mechanism would be required.

The complexity of the response of *S. stapfianus* to dehydration/desiccation and rehydration was evident from the many changes in both individual metabolite and transcript abundances, which as discussed by Dinakar and Bartels 2013 [7], this requires a “fine-tuned regulatory network”. The complexity of these responses is also reflected in the heterogeneity of the transcripts for regulatory proteins that change significantly in abundance during dehydration. In the early stages of dehydration, between Hydrated and 80% RWC, transcripts encoding protein phosphatase 2C proteins, a negative regulator of ABA signaling in the acquisition of desiccation tolerance [75], in combination with low levels of ABA at these leaf water contents [13] suggest that ABA does not control the initial response to dehydration. The decrease in the abundance of these transcripts prior to the increase in endogenous ABA concentrations possibly reflects the importance of this phytohormone in the latter stages of drying as desiccation tolerance is acquired. However, the transcriptome results do point to signaling pathways that might be required for responding to dehydration and preparing for desiccation. An increase in the abundance of transcripts encoding phospholipase D indicates the early activation of a non-ABA signal transduction pathway similar to that described for the dehydration response to of the resurrection dicot *Craterostigma plantagineum* [76]. In addition, the early increase in the abundance of transcripts encoding SNF1-related protein kinase regulatory subunit gamma-1-like proteins (gamma-SnRK1), which are required for stability and substrate specificity [77], suggests that the rapid change in carbohydrate (particularly sucrose) concentrations and energy status of *S. stapfianus* leaves early in the dehydration event [12] might also play an active role in the stress signaling pathways required to prepare for desiccation. Calcium signaling also appears to play a role in orchestrating both the dehydration and the later desiccation stages of leaves as transcripts for calcineurin B-like (CBL) proteins accumulate both early, between Hydrated and 80% RWC, and late as leaves reach 30% RWC. CIPK-like protein 1 transcripts, kinases that interact with CBL proteins [78], also accumulated late in the drying process. The complexity of the signaling response is also reflected by the fact that many transcripts that are annotated as encoding the same signaling protein are represented by different contigs and exhibit both increases and decreases in abundance. This suggest that these contigs represent transcripts from different members of a gene family or different splicing events that are regulated in different manners. Such regulation might occur in a tissue/cell specific manner and, as the whole leaf is used to generate an RNA sample, this possibility could not be interrogated.

The complexity of the signaling response is also apparent in the changes in transcript abundance of a variety of transcription factors (TF) that alter gene expression and thereby orchestrate the dehydration response. The expression of transcripts for over 60% of the TFs identified in the 454-derived leaf transcriptome were negatively impacted by dehydration. Presumably, the majority of these TFs, such as those involved in auxin-mediated responses, were associated with normal growth, differentiation, and development, which ceases as the plant dehydrates. The expression of TFs involved in regulating the circadian clock and flowering is also repressed by dehydration. Surprisingly however, the transcriptome did not contain an induced SDAT representing the AP2/ERF family of transcription factors, particularly a dehydration responsive element binding (DREB) factor, as these are known to participate in dehydration responses in non-desiccation tolerant plants [79] and the resurrection shrub *Myrothamnus flabellifolia* [80]. The abundance of such DREB transcripts might be too low to have been represented in the *S. stapfianus* 454-generated transcriptome, but no responsive DREB factor appeared in the dehydration transcriptome of the resurrection plant *Craterostigma plantagineum* either [50]. Thus, perhaps DREB factors are not necessary for the regulation of desiccation tolerance in all resurrection species. The abundance of the majority of the TFs classified as positive SDATs increased relatively early in the dehydration process and peaked when leaves reached 30% RWC. The earliest TFs to peak in abundance were a zinc knuckle C3H protein (Contig 373) and a homeobox domain leucine zipper (HD-Zip) protein (Contig 32671) that peaked at 60% RWC. Zinc finger C3H proteins have been implicated in salt/dehydration stress responses in cotton [81] and in stress tolerance in transgenic *Arabidopsis* by regulating ABA/sugar responses [82]. The expression of *OsZFP6*, a rice CCHC-type zinc finger protein, is regulated by several types of stress treatments, in particular oxidative stress, and overexpression of this protein in *Arabidopsis* rendered the transgenic plant more tolerant to hydrogen peroxide treatment [83]. The *Arabidopsis* HD-Zip transcription factor homologs ATHB1 and ATHB10 function in environmental and hormonal signal transduction pathways, respectively [84]. Along with the early responsive transcript encoded by Contig 3267, we identified three other contigs (1803, 1804, and 16051) encoding HD-Zip proteins whose transcripts all peaked in abundance late during drying, concomitant with the peak in ABA abundance (Fig. 6). Five HD-Zip transcription factors were identified in the resurrection dicot *Craterostigma plantagineum* all of which responded to dehydration and ABA, but were repressed upon rehydration [85]. One of these *Craterostigma* HD-Zip proteins (CpHB-7) acted as a negative regulator of ABA-responsive gene expression when expressed ectopically in tobacco [86]. An ABRE binding factor transcript also accumulated early in dehydration, but peaked late as the leaves approached 30% RWC, and acted as a positive regulator of ABA biosynthesis [87]. Such an increase coincides with induction of several ABA-responsive genes, as discussed earlier. Similarly, the abundance of transcripts encoding up to four dehydration-inducible SDATs encoding HSF-TF proteins, all increased during dehydration, coincident with the induction of several small HSPs and molecular chaperones. Other TF SDATs, such as those encoding NF-Y, NAC, and bZIP proteins, have also been induced by dehydration in other resurrection plants [7], although their possible target genes related to desiccation tolerance are unknown.

### The rehydration and recovery response

Rehydration from a fully dried state is as stressful to plant tissues as dehydration, on structural to physiological and biochemical levels [88]. Although rehydration from the dried state elicits a significant cellular response in DT bryophytes [89], it is somewhat surprising that resurrection angiosperms exhibit a more muted response ([90]; this report). This may simply reflect a more effective cellular protection mechanism in the resurrection angiosperms that severely limits desiccation-induced damage, but nevertheless, rehydration-induced mechanisms are likely critical for the recovery of growth when full water status is restored [90].

In *S. stapfianus*, rehydration-specific responses are limited both metabolically and transcriptionally as compared to the dehydration response. As in other resurrection species, the major response is to quickly return to the pre-desiccation hydrated status. In *S. stapfianus*, the responses of both the metabolome and the transcriptome indicated that recovery was not fully achieved within the 24 h rehydration period used in our study. Rehydration *per se* can take some time, even though the rehydration procedure we use applies water to both the soil and aerial portions of the plant. Rehydration may also involve rapid recovery of aquaporins, or plasma membrane intrinsic proteins (PIPs), to facilitate water movement into cells as evidenced by rapid recovery in the abundance of transcripts encoding PIPs within the first 12 h of rehydration (Contigs 3018, 23020, and 23104, Additional file 4: Table S4). Notably, transcripts encoding tonoplast membrane intrinsic proteins (TIPS) accumulate, presumably to regulate water movement in and out of the vacuoles (contigs 6770 and 6771).

Photosynthesis, as measured by CO_2_ assimilation recovers rapidly and reaches approximately 70% of control hydrated values within the first 24 h after rehydration [13]. The recovery of photosynthesis was reflected in these data by the rapid re-accumulation of transcripts encoding enzymes and components of the photosynthetic machinery and associated metabolism, most notably transcripts encoding Chlorophyll a/b binding (CAB) proteins and PEPC (Additional file 5: Table S5). Metabolic intermediates in the C_4_ carbon assimilation pathway and the Calvin Cycle were generally at too low concentrations to be reliably detected in our experiments, with the exception of malate, pyruvate, oxaloacetate, and fructose-6-phosphate. Both pyruvate and oxaloacetate remain depleted during rehydration, but malate levels, which were elevated in the dried leaves, remained above the hydrated control levels. The inference is that PEPC activity did not fully recover after 24 h of rehydration and that the decarboxylating activity of malate dehydrogenase (NADP+) might also be inhibited. The accumulation of fructose-6-phosphate might indicate the recovery of the Calvin Cycle, but also coincides with similar accumulation of glucose-6-phosphate, which might indicate alterations in glycolytic activity as the leaves recover. Upon rehydration, respiration rates also recover, increasing linearly with increasing relative leaf water content [91] also noted that sucrose was not mobilized in the early stages of rehydration and that alternate substrates such as amino acids were presumably used for respiration. The metabolome presented here supports that conclusion, as sucrose levels remained elevated, near the level recorded for dried leaves, and did not drop significantly during 24 h of rehydration (Fig. 7). ATP is retained in dried leaves of *S. stapfianus,* but at reduced levels relative to the hydrated state, but perhaps sufficient to fuel the initial stages of rehydration [91]. Nevertheless, the abundance of transcripts encoding ATP synthase recovered rapidly (Additional file 5: Table S5c).

The abundance of several amino acids either remained elevated or became elevated upon and during rehydration, perhaps reflecting the slow return to normal growth and nitrogen assimilation patterns. *S. stapfianus* is a slow-growing grass even under well-watered conditions and thus is expected to return slowly to normal growth rates following a desiccation event. Two important kinetic factors are associated with the return of amino acid pools to the normal hydrated control state. One is the synthesis of proteins required for recovery or to replenish proteins depleted under desiccation. The other is the turnover of damaged proteins to restore protein homeostasis. The rehydration transcriptome reflects the activity of these two processes in the first 24 h of rehydration as increases in the abundance of transcripts associated with both protein synthesis and turnover to control or higher levels. Transcripts for translation initiation and elongation factors and a subunit of the peptide release factor are both prominent in the rapid recovery of transcripts encoding proteins involved in protein synthesis. Similar increases in abundance of transcripts involved in protein synthesis upon rehydration were reported for the resurrection shrub *Myrothamnus flabellifolia* [80] indicating that a rapid return of protein synthetic activity is a common feature of resurrection plant dehydration tolerance mechanisms. Earlier work by O’Mahony and Oliver (1999) [92] suggested an important role for ubiquitin in the response of *S. stapfianus* to rehydration, presumably related to its role in protein turnover. An increase in transcripts for polyubiquitin as well as increases in both ubiquitin monomer levels and ubiquinated proteins occur during rehydration. The leaf transcriptome reinforces the importance of the role of protein ubiquitination during rehydration: the abundance of ubiquitin transcripts recovers to levels above those of controls and the abundance of ubiquitin-activation and ubiquitin conjugation protein transcripts also recovers rapidly (Additional file 6: Table S6i). Also, the need to turn over damaged proteins upon rehydration could account for the increase in concentrations of some amino acids in the first 24 h after the re-addition of water.

The rehydration metabolome indicates the need to protect membranes from ROS cellular homeostasis is reestablished, as reflected by the maintenance of increased levels of the lipophylic antioxidants alpha-, beta-, and delta-tocopherol. Tocopherols prevent the proliferation of lipid peroxidation in membranes [93], which constitutes a serious threat to membrane integrity. The reported elevation in the content of lysolipids in dried leaf tissue of *S. stapfianus* indicates that lipid peroxidation does occur during dehydration [12] and although the levels of lysolipids then decline during rehydration (Additional file 1: Table S1) the potential for peroxidation still exists. The generation of ROS and their effects on membrane integrity, have long been associated with rehydration (or imbibition) of desiccation-tolerant tissues [88] Together with the maintenance of high levels of tocopherols, *S. stapfianus* also maintained elevated levels of the cytoplasmic antioxidant, reduced glutathione. The rehydration transcriptome emphasizes the continued importance of protecting tissues from oxidative metabolism and ROS during rehydration through the elevated abundance of transcript for several enzymes involved in redox homeostasis, including catalase and phospholipid hydroperoxide glutathione peroxidase.

The complex control of gene expression associated with rehydration appears to be as intricate as that involved in the response to dehydration. Just as for dehydration, there were indications in these experiments that both ABA and calcium-signaling pathways are important in the response to rehydration. This was exemplified by the accumulation of transcripts encoding an ABA-activated serine/threonine-protein kinase SAPK6, protein phosphatase 2C, and CBL-interacting protein kinases (as discussed earlier). Serine/threonine-protein kinase SAPK6 phosphorylates bZIP transcription factors that regulate downstream gene expression events associated with responses to water-deficit stress and are inactivated by protein phosphatase 2C [94]. In concordance with the transcriptome, the elevated endogenous ABA levels (Fig. 6) in the rehydrating leaves suggest that the ABA signaling pathway is up-regulated during the recovery process. The possibility that ABA might actually play a role in the control of gene expression upon rehydration is also suggested by the increase in abundance of transcripts encoding several TFs that are often targets for ABA signaling pathways. The zinc finger A20 and AN1 domain-containing stress-associated protein is reportedly induced by drought and ABA [95], as are the NAC type BTF3 transcription factors [96]. It is also noteworthy that transcripts for a two-component sensor histidine kinase accumulate during the rehydration process, as these proteins are often associated with plant responses to environmental changes in and have been closely linked to redox sensing under abiotic stress [97].

## Conclusions

The combination of transcriptome and metabolome analyses has revealed further insights into the complexity of the desiccation response and the vegetative desiccation tolerance trait in the C_4_ grass *Sporobolus stapfianus*. The concordance between the transcriptome and metabolome appears to be relatively strong in areas of this response related directly to cellular protection during dehydration. This is particularly evident in the dehydration-induced accumulation of transcripts for enzymes that synthesize carbohydrates (primarily sucrose, raffinose, and stachyose), whose peaks in abundance just precede those of their carbohydrate metabolites (Fig. 4 and [12]). The concordance between transcript and metabolite abundance also appears to hold for some of the key anti-oxidants such as glutathione and tocopherols. There is almost no concordance between transcript and metabolite abundance for processes that do not appear to contribute directly to cellular protection, but are important for the DT phenotype of *S. stapfianus.* One example is the lack of a transcriptional response related to the accumulation of amino acids in young DT leaves as water is lost from the plant. Although the retention and storage of nitrogen as amino acids for remobilization during rehydration might be critical for the survival of *S. stapfianus* after the end of a dry period, it does not appear to play a major role in the protection of the cells during the later stages of drying and in the dried state. The accumulation of amino acids during the latter stages of drying, although reported for both *S. stapfianus* (here and [15]) and *Selaginella lepidophylla* [74], is not a universal phenotype in resurrection species [7]. The metabolomic and transcriptomic data support the hypothesis that the build-up of amino acids in the leaves of *S. stapfianus* during drying results from the mobilization of nitrogen from the mature DS leaves that senesce during this process, as proposed by Martinelli et al, 2007 [15]. This might be a survival feature derived from the perennial aspects of nitrogen storage and remobilization during overwintering commonly seen in C_4_ forage grasses [16]. This would allow *S. stapfianus* to make efficient use of assimilated nitrogen during rehydration and is perhaps a critical need in its native nutrient-poor habitat [98]. Much of the dehydration transcriptome appears to support the metabolic focus of cellular protection, with increases in the abundances of LEAs, ELIPs, ROS protection enzymes, and cell wall enzyme transcripts.

It is difficult to determine if transcript and metabolite abundance are coordinated during rehydration, as we do not have sufficient time points to make a reliable determination. The general trend during rehydration is a rapid return to normal metabolite and transcript abundance levels, with only a few metabolites, primarily involved in ROS protection, displaying increases in abundance. Overall, the rehydration transcriptome is geared towards the rapid return of photosynthesis, energy metabolism, protein turnover, and protein synthesis.

The transcriptomes of both dehydration and rehydration offer insight into the complex regulation of the response to both environmental perturbations. The early response to dehydration appears to be controlled by non-ABA signaling mechanisms, similar to those seen in the desiccation-tolerant dicot *Craterostigma plantagineum* [76], that include the phospholipase D, calcium, and sugar signaling pathways. ROS signaling might also play a key role in the control of the transcriptomic response to dehydration. The responses to both dehydration and rehydration involve the activity of a myriad of TFs spanning most known TF gene families. ABA, as reported earlier [13], appears to play a major role in the latter stages of the dehydration response as desiccation is approached. However, ABA levels remain high during the early stages of rehydration, concomitant with an increase in the abundance of transcripts of ABA-regulated genes, including ABA-responsive TFs, implying a role for this hormone in the rehydration response. The high level of ABA in rehydrating tissue might simply reflect the fact that the rehydration process is incomplete and there is still considerable dehydration stress in the young leaves, even 24 h after the plant received water. It might also reflect a need for transcripts involved in redox homeostasis and protein turnover.

## Methods

### Plants and drying treatment

*Sporobolus stapfianus* Gandoger (original provenance: Verena, Transvaal, South Africa) originally obtained from the collection of Dr. Don Gaff, Monash University, Victoria, Australia who originally identified and collected them. Seeds are currently vouchered at the University of Missouri seed storage facility curated by the United States Department of Agriculture, Agricultural Research Service, Plant Genetics Research Unit. Plants were grown, maintained, and seed stocks increased (as described by O’Mahony and Oliver (1999 [92]), in one-gallon pots under greenhouse conditions (16-h light and day/night temperatures of 28°C/19°C). Three-month-old plants for each experiment (metabolomics, RNA isolation for Niblegen array analysis, and endogenous ABA) were subjected to a drying event under greenhouse conditions by withholding water. Young leaf tissue was collected at regular daily intervals, between 9 and 10 am, from individual plants and flash-frozen in liquid N_2_ and stored at -80 °C. Dried plants were maintained dry for a week before rehydration. Duplicate samples were for RWC measurements of the plant at time of sampling. RWC was calculated as the formula (Fwt 2 Dwt) (FTwt 2 Dwt), where Fwt was the fresh sample weight, FTwt was the full turgor weight of the same sample after submersion in deionized water overnight in the dark, and Dwt was the weight of the sample after being dried to equilibrium at 70°C for 4 h. All RWC values given in the text are plus or minus 2%. Rehydration was achieved by placing the desiccated plants under a continuous misting system under greenhouse conditions and young leaves were samples at 12h and 24h.

### Metabolomic profiling platform

Young leaf tissue was collected from each plant and flash frozen in liquid nitrogen and stored at -80°C. Six replicate samples, from individual plants, were collected for each dehydration step representing fully hydrated tissue (HYD), 60%, 40, 30% RWC, and desiccated (DRY), and rehydrated (12 h and 24h). samples (six replicates per treatment) used for the analysis of the rehydration metabolome were identical to those reported in the earlier study [12]. The global unbiased metabolic profiling platform, metabolite analysis, and statistical treatments of the metabolite data was as described in Oliver et al., 2011 [12]. In brief, the statistical analysis was performed using JMP [99], a commercial software package, and R (http://cran.r-project.org/), an open-source software package. A log transformation was applied to the relative concentrations for each metabolite because the variance generally increased as a function of each metabolite’s average response. Welch’s t tests, a variation of Student’s *t* test for samples with unequal variances, were used to compare data between experimental groups (see Oliver et al., 2011 [12] and Additional file 1: Table S1).

### Endogenous ABA

Eight replicate samples, from individual plants, were collected for each dehydration step representing fully hydrated tissue (HYD), 60%, 40, 30%, and desiccated (DRY), and rehydrated (12 h and 24h). samples (eight replicates per treatment) used for ABA extraction. Abscisic acid was extracted from lyophilized tissues according to a method developed from Chiwocha et al. (2003) [100] with some modifications. Briefly, leaf samples were flash-frozen and ground into a fine power in liquid N_2_ before freeze-drying. Twenty milligrams of tissue from each freeze-dried sample were placed in a 2-ml tube and spiked with 5 ng/ml of d6-ABA (internal standard, 0.1 mg/ml) before extraction with 1 ml of 80% isopropanol containing 1% glacial acetic acid by vigorous vortexing for 1 min. Each sample was agitated at 4 °C for 24 h in the dark and spun at 14,000 rpm for 15 min at 4 °C. The supernatant for each sample was transferred to a clean tube placed on ice. The pellets were reconstituted and the extraction procedure was repeated twice with 0.5 ml of the same solvent, and centrifuged for 5 min. The three supernatants were combined and filtered with 0.2 um Whatman Anatop Disposable Syringe Filters (Whatman, Inc., Florham Park, NJ, USA) and evaporated to dryness in a SpeedVac Concentrator set to 46°C.

Freeze-dried extractions were reconstituted with 80 μl of a mixture of the two mobile phases that consist of water containing 1% acetic acid (A) and acetonitrile containing 1% acetic acid (B). The analysis was performed using an Agilent 1260 LC equipped with a quaternary pump, a thermostat column compartment, and an auto-sampler coupled with a single quadrupole mass spectrometer (MS) equipped with electronic ionization mode. ABA was separated using ZORBAX Eclipse Plus C18 (150 x 4.6 mm; 3.5 μm) column maintained at 30 °C. The injection volume was set to 40 μl and the flow rate was set to 0.4 ml/min. The mobile phase gradient was as follows for B: 15% for 0-10 min; 35% for 14.2 min; 40% for 15–18.2 min; 60% for 18.3–18.4 min; 62% for 20–25 min; and 15% for 26–35 min. For the MS, the drying gas flow was set to 9.5 l/min, the nebulizer pressure to 45 psig, and the drying gas temperature to 350 °C. The acquisition mode was set to SIM negative with 263 and 264 ions targeted for ABA and 269 and 270 ions targeted for ABA-6d. The fragmenter was set to 90 V and the gain was set to 1.

An ABA standard curve, standard stock solutions of (+/-)-cis, trans-abscisic acid (Sigma-Aldrich, Co. LLC., Saint Louis, MO, USA) and (+)-cis, trans- abscisic acid-d6 (Santa Cruz Biotechnology, Inc., Dallas, TX, USA) were prepared at a concentration of 0.1 mg/ml in 70% methanol before serial dilution with 70% methanol to obtain working solutions. Ten microliters of each working solution and internal standard (d6-ABA, 0.1 mg/ml) were added to 20 mg of freeze-dried control leaf tissue (non-stressed) samples and extracted with 990 ml of 80% isopropanol containing 1% glacial acetic, as described earlier in this section. The calibration curve was prepared using seven calibration standards with final ABA concentrations of 0.5, 1, 2, 5, 10, 25, and 50 ng/ml. Control samples spiked with 5 ng/ml of internal standard (d6-ABA, 0.1 μg/ml were extracted in parallel to account for the contribution of endogenous ABA. The calibration curve was constructed by plotting the peak area ratio of endogenous ABA to internal standard (d6-ABA) against the concentration in the standard-spiked control sample by least-squares linear regression (*R* ^2^ = 0.99883).

### Plant Material and mRNA Isolation

*Sporobolus stapfianus* plants used for 454 sequencing and microarray analysis are the same as those described in Oliver et al. (2011) [12], Monash University. Total RNA was isolated from triplicate samples of young leaves, each sample composed of pooled tissue from three individual plants, of fully hydrated (HYD) 96% RWC), dehydrating 80%, 60%, 40%, 30% RWC, and desiccated (DRY) 11% RWC, and rehydrated (12 h and 24h) plants using RNeasy Mini Kit extraction (Qiagen, Inc., Valencia, CA) according to the manufacturer’s instructions. The water contents (RWC vs absolute water content) are described in the drying curves reported by Oliver et al., 2011. After electrophoretic analysis on formaldehyde-containing 1.2% agarose gel and quantification (NanoDrop ND-1000 UV-Vis spectrophotometer; NanoDrop Technologies Inc., Rockland, DE, USA), equal amounts of RNA were pooled and mRNAs were isolated using a PolyATract mRNA Isolation System III according to the manufacturer’s instructions (Promega, Madison, WI, USA).

### 454 Life Sciences (Roche) Pyrosequencing

A cDNA library was constructed using *S. stapfianus* mRNA as template to synthesize first-strand cDNA using a Modified Smart cDNA Synthesis Kit from Clontech Laboratories, Inc. (Takara Bio Company, Mountain View, CA, USA) according to the manufacturer’s instructions. The primers used were: 5’-AAGCAGTGGTATCAACGCAGAGTGGCCATTACGGCC-GGG-3’ for Smart IV, and 5’-TAGAGACCGAGGCGGCCGACATGTTTTGTTTTTT-TTTCTTTTTTTTTTVN-3’ for modified CDS III/3’. The second-strand cDNA was synthesized using the primer extension method (Clontech Laboratories, Inc.). Following gel electrophoresis of 5-μL samples, the remaining 95 μL of the second-strand product was applied to a Clontech TE-400 size-fractionation column (Clontech Laboratories, Inc.) as instructed by the manufacturer. The recovered product was then cleaned up (using Qiagen kit), eluted in 30 μL of nuclease-free water, and quantified by spectrophotometer (NanoDrop Technologies, Inc.). The cDNA was treated to generate blunt ends and ligated to 454-specific adaptors, then small fragments were removed using AMPureXP Beads (Agencourt Bioscience Corporation, Beverly, MA). The library was normalized to remove high-abundance cDNAs using the Trimmer kit (Evrogen, City, Russia). The cDNA was then quantified using a Qubit fluorometer (Invitrogen, Inc., City, CA, USA) and average fragment sizes were determined using a Bioanalyzer (Agilent, CA). The cDNA was diluted to 1 × 10^6^ molecules/μl and emulsion-based amplification and sequencing on the 454 Genome Sequencer (GS)-FLX Titanium system according to the manufacturer’s instructions (Roche Applied Sciences, Indianapolis, IN). Signal processing and base calling were performed using the bundled Roche 454 Data Analysis Software resulting in 490,144 clean reads. The *de novo* assembly of the 454 reads was performed using GS De Novo Assembler software version 2.8, in cDNA assembly mode, minimum overlap length of 40 nt, minimum match identity of 95%. Sequencing and *de novo* assembly were performed by the Michigan State University Research Technology Support Facility [101]. Cleaned 454 sequences were deposited in the NCBI Database [102] as a Sequence Read Archive, reference number SRR5329400, and the contigs as a Transcriptome Shotgun Assembly project that has been deposited at DDBJ/EMBL/GenBank under the accession GFJP00000000. The version described in this paper is the first version, GFJP01000000.

### Microarray fabrication and hybridization design

A custom microarray was made using the fabrication and design services of Roche NimbleGen [103]. The array contained gene models for 50,690 contigs and controls, each of which was represented by 7 individual 60-mer homologous probes. For hybridizations, total RNA was extracted from young leaf tissues of plants at 3, 2, 1.5, 1, 0.75, and 0.25 g H_2_O/g tissue, as well as of plants rehydrated for 12 or 24 h. RNAs were sent to Roche NimbleGen, where cDNA synthesis, biotin-labeling, and hybridization were performed according to the manufacturer’s protocols. Briefly, total RNA (15 μg) from three biological replicates, each replicate consisting of pooled tissue from three individual plants, of fully hydrated (96% RWC), dehydrating 80%, 60%, 40, 30% RWC, and desiccated 11% RWC, and rehydrated (12 h and 24h) for a total of 24 samples, were converted to biotin-labeled cRNA using the SuperScript double-stranded cDNA Synthesis Kit (Invitrogen), labeled with a NimbleGen One-Color DNA Labeling Kit, and hybridized to arrays using NimbleGen hybridization platform (Roche NimbleGen). Array scanning was performed using the GenePix 4000B Microarray Scanner and data were collected using Roche NimbleGen software.

### Gene expression analysis

The NimbleGen custom oligonucleotide array images were first examined visually in their raw data format for gross spatial variation due to fibers or bubbles. Raw array data were processed and normalized by Robust Multi-Array Average (RMA) [104] using the R package affy [105]. Expression values were computed by applying the RMA model of probe-specific correction of perfect match probes. The processed probe values were then normalized via quantile normalization, and a median polish was applied to compute one expression measure from all probe values.

Data were then cleansed to exclude highly outlying replicate expression values. Any set of triplicates with a coefficient of variation (CV) greater than 0.5 and a replicate value with a standard deviation greater than 1 across the three replicate expression values was further scrutinized. The maximum possible standard deviation for three measures in this data set was 1.15; if one of the three replicates was more than one standard deviation away from the mean triplicate expression value, this indicated a very high probability (98.5%) that the remaining two measures were very similar, and that the removal of this replicate would decrease the CV across the three replicates.

Of the 400,856 sets of triplicates, 10% exhibited a CV greater than 0.50. Only 39,492 expression values (3.3% of all measures generated) were excluded as single outliers, and 2,214 (0.18%) were excluded as complete replicates, due to extremely high (>.75) CV that could not be moderated by the removal of any single outlying value. The remaining 1,160,862 (96.5%) values had an average CV of 0.18, typical of the microarray experiments processed by the Nevada INBRE Bioinformatics Core. These thresholds permit identification of gross outlying individual measurements within a replicate set, as described previously [106]. The complete dataset was deposited in the NCBI Gene Expression Omnibus data store and can be accessed after March 1^st^ 2017 [107].

#### Statistical analysis

A simple one-way ANOVA [108] was performed on the normalized and cleansed data to determine which features on the array were differentially expressed across the hydration levels. A multiple testing correction was applied to the p-values of the ANOVA [109], and any feature with a significant term with adjusted *p*-value *p* < 0.05 was deemed statistically significant. A total of 4,739 transcripts were identified as significantly differentially expressed using this process.

Because our dehydration treatments were based relative water contents and our rehydration treatment was based on the time after rewatering, the experimental data were separated into two groups during the analysis. The first group consists of six experimental conditions (Hydrated [HYD] 96%, 80, 60, 40, 30, and 11% RWC [DRY]), whereas the second group consists of three experimental conditions (DRY, 12 h rehydrated and 24 h rehydrated). Each group was then clustered using a simple hierarchical clustering procedure, in which clusters were determined by using an average pairwise Pearson correlation measure of 0.85 or greater. Notably, 171 genes were excluded from Group 2 due to not having enough values to compare after the cleansing procedure described above.

### Data annotations and functional analysis

The 50,690 assembled contigs were used as a blast query against the non-redundant (nr) GenBank, UniProt, and *Arabidopsis* protein databases with a cutoff value of < 1e-5 [110]). InterProScan (IPS). A Blast2GO [111] integrated tool, was used to identify domain signatures [112] and GO terms for each contig were mapped to the InterPro entries.

The Cytoscape plugin tool BiNGO was used to determine enrichment of SDATs. A hypergeometric test with at *p*-value < 0.05 after applying a Benjamini-Hochberg False Discovery Rate (FDR) correction for multiple comparisons [108] was considered significant. AgriGO, a publicly available resource, was used to further determine the enrichment of all GO terms: biological process, molecular function, and cellular component [113]. Heat maps were generated using the web-based open source software; Multi Experiment Viewer (MeV 4.8.1) (Saeed et al., 2003) [114].

### Quantitative Real-Time (qRT)-PCR Analysis

For microarray data validation, total RNA was extracted from 18 leaf samples, each from an individual plant treated as described above, representing six hydration states (HYD, 80%, 60, 40, 30% RWC, or Dry tissue) in triplicate. For each sample, 3 μg of total RNA was used to synthesize the first-strand cDNA that was used to amplify 10 genes identified by analysis of the microarray data. Primer design was performed using the MacVector 11.0.4 Primer3 program (MacVector, Inc., Cary, NC, USA). Reactions were conducted on a 7900 HT Fast Real-Time PCR System (Applied Biosystems Inc., Foster City, CA, USA) using the SYBR® Green Master Mix (Applied Biosystems, Inc.) reagent in a 10-μl volume. PCR conditions were as follows: 95 ^o^C for 10 min followed by 30 cycles of 95 ^o^C for 15 sec and 60 ^o^C for 1 min. Relative quantification was performed using the ∆∆Ct method [115]. The list of the primers used is presented in Additional file 2: Table S2. To assess the validity of the microarray generated patternsof transcript abundance the log2 ratios of 80% RWC/HYD, 60% RWC/HYD, 40% RWC/HYD, 30% RWC/HYD and DRY/HYD were compared between the PCR and the microarray data. A simple linear regression showed a relatively good fit between the two technologies, with R^2^ = 0.843. The hypothesis test of a zero slope was rejected with a *p*-value of p < 1 × 10^−15^. Additionally, the Spearman correlation coefficient between the two sets of expression values was computed to measure correlation as these expression data were not perfectly normally distributed. The Spearman correlation coefficient was measured as 0.901, again showing a good correlation between the two platforms.

## Additional files

Additional file 1: Table S1. Non-targeted global metabolite profiles of dehydrating and rehydrating *Sporobolus stapfianus* leaf tissues. (XLSX 190 kb)

Additional file 2: Table S2. Gene Ontology (GO) enrichment of SDAT transcripts associated with the dehydration and rehydration of young leaves of *Sporobolus stapfianus*. (XLSX 24 kb)

Additional file 3: Table S3. InterProScan protein signatures generated annotation for assembled contigs. (XLSX 306 kb)

Additional file 4: Table S4a. ANOVA analysis and log^2^ expression values for *Sporobolus stapfianus* leaf SDATs during both dehydration and rehydration. **Table S4b.** Fold change (log_2_) in abundance and cluster assignment for SDATs during dehydration of young leaves of *Sporobolus stapfianus*. **Table S4c.** Fold change (log^2^) and cluster assignment for SDATs during rehydration of young leaves of *Sporobolus stapfianus. (XLS 1622 kb)*

Additional file 5: Table S5a. A representative list of SDATs that have the greatest increase in abundance during dehydration of young leaves of *Sporobolus stapfianus*. **Table S5b.** A representative list of SDATs that have the greatest decrease in abundance during dehydration of young leaves of *Sporobolus stapfianus*. **Table S5c.** A representative list of SDATs that have the greatest increase in abundance during rehydration of young leaves of *Sporobolus stapfianus*. **Table S5d**. A representative list of SDATs that have the greatest decrease in abundance during rehydration of young leaves of *Sporobolus stapfianus*. (XLS 78 kb)

Additional file 6: Table S6a. SDATs encoding antioxidant enzymes and enzymes involved in antioxidant biosynthesis. The color shading represents a statistically significant change in transcript abundance in the dehydrated samples ((80%, 60, 40, and 30% RWC) from the abundance of the transcript in the hydrated control (HYD). In the rehydrating samples (12h and 24h), the color shading represents a significant change in transcript abundance from that of the hydrated control (HYD) or the dried samples (DRY). Red indicates a statistically significant increase in transcript abundance and green a statistically significant decrease in abundance. **Table S6b.** SDATs encoding enzymes involved in carbohydrate and energy metabolism. The color shading represents a statistically significant change in transcript abundance in the dehydrated samples (80%, 60%, 40%, and 30% RWC) from the abundance of the transcript in the hydrated control (HYD). In the rehydrating samples (12 h and 24 h), the color shading represents a significant change in transcript abundance from that of the hydrated control (HYD) or the dried samples (DRY). Red indicates a statistically significant increase in transcript abundance and green a statistically significant decrease in abundance. **Table S6c.** SDATs encoding enzymes involved in cell wall biosynthesis and catabolism. The color shading represents a statistically significant change in transcript abundance in the dehydrated samples (80%, 60, 40, and 30% RWC) from the abundance of the transcript in the hydrated control (HYD). In the rehydrating samples (12 h and 24 h), the color shading represents a significant change in transcript abundance from that of the hydrated control (HYD) or the dried samples (DRY). Red indicates a statistically significant increase in transcript abundance and green a statistically significant decrease in abundance. **Table S6d.** SDATs encoding kinases and phosphatases. The color shading represents a statistically significant change in transcript abundance in the dehydrated samples (80%, 60%, 40%, and 30% RWC) from the abundance of the transcript in the hydrated control (HYD). In the rehydrating samples (12 h and 24 h), the color shading represents a significant change in transcript abundance from that of the hydrated control (HYD) or the dried samples (DRY). Red indicates a statistically significant increase in transcript abundance and green a statistically significant decrease in abundance. **Table S6e.** SDATs encoding transcription factors. The color shading represents a statistically significant change in transcript abundance in the dehydrated samples (80%, 60%, 40%, and 30% RWC) from the abundance of the transcript in the hydrated control (HYD). In the rehydrating samples (12h and 24h), the color shading represents a significant change in transcript abundance from that of the hydrated control (HYD) or the dried samples (DRY). Red indicates a statistically significant increase in transcript abundance and green a statistically significant decrease in abundance. **Table S6f.** SDATs encoding late embryogenesis abundant (LEA) proteins. The color shading represents a statistically significant change in transcript abundance in the dehydrated samples (80%, 60, 40, and 30% RWC) from the abundance of the transcript in the hydrated control (HYD). In the rehydrating samples (12 h and 24 h), the color shading represents a significant change in transcript abundance from that of the hydrated control (HYD) or the dried samples (DRY). Red indicates a statistically significant increase in transcript abundance and green a statistically significant decrease in abundance. **Table S6g**. SDATs encoding heat shock and molecular chaperones. The color shading represents a statistically significant change in transcript abundance in the dehydrated samples (80%, 60, 40, and 30% RWC) from the abundance of the transcript in the hydrated control (HYD). In the rehydrating samples (12 h and 24 h), the color shading represents a significant change in transcript abundance from that of the hydrated control (HYD) or the dried samples (DRY). Red indicates a statistically significant increase in transcript abundance and green a statistically significant decrease in abundance. **Table 6h**. SDATs encoding ABA-induced transcripts. The color shading represents a statistically significant change in transcript abundance in the dehydrated samples (80%, 60%, 40%, and 30% RWC) from the abundance of the transcript in the hydrated control (HYD). In the rehydrating samples (12 h and 24 h), the color shading represents a significant change in transcript abundance from that of the hydrated control (HYD) or the dried samples (DRY). Red indicates a statistically significant increase in transcript abundance and green a statistically significant decrease in abundance. **Table S6i**. SDATs encoding proteins and enzymes involved in protein biosynthesis and turnover. The color shading represents a statistically significant change in transcript abundance in the dehydrated samples (80%, 60, 40, and 30% RWC) from the abundance of the transcript in the hydrated control (HYD). In the rehydrating samples (12 h and 24 h), the color shading represents a significant change in transcript abundance from that of the hydrated control (HYD) or the dried samples (DRY). Red indicates a statistically significant increase in transcript abundance and green a statistically significant decrease in abundance. (XLS 308 kb)

Additional file 7: Figure S1. Predominant clusters of SDATs that share distinct patterns of abundance during dehydration: A. Predominant patterns of abundance for transcripts in clusters that exhibited increased abundance during dehydration. B. Predominant patterns of abundance for transcripts in clusters that exhibited a decreased abundance during dehydration. (PDF 226 kb)

Additional file 8: Table S8. Expression analysis raw data listing both qPCR and Array values for each gene used in the validation of the array expression analysis. (XLSX 11 kb)

Additional file 9: Table S7. A list of the primers used for the quantitative real-time PCR used to validate the microarrays derived transcript abundance values. (XLSX 34 kb)

## Abbreviations

γ-GCS: γ-glutamyl cysteine synthetase
13-HODE: 13-hydroxy-9,11-octadecadienoic acid
9-HODE: 9-hydroxy-10, 12-octadecadienoic acid
ABA: Abscisic acid
ATP: Adenosine triphosphate
BiNGO: Biological Network Gene Ontology
CAB: Chlorophyll a/b binding
CBL: Calcineurin B-like
cGMP: Guanosine-3’,5’-cyclic monophosphate
CV: Coefficient of variation
DREB: Dehydration responsive element binding
DS: Desiccation sensitive
DT: Desiccation tolerance
ELIP: Early light inducible
EST: Expressed sequence tag
GABA: Gamma-aminobutyrate
GGT: Gamma-glutamyl transpeptidase
GO: Gene ontology
GPC: Glycerophosphorylcholine
GR: Glutathione reductase
GS: Glutathione synthase
HD-Zip: Homeobox domain leucine zipper
IAA: Indole acetic acid
LEA: Late embryogenesis abundant
MPa: Megapascals
NCBI: National Center for Biotechnology Information
OAA: Oxaloacetate
PEPC: Phospho*enol*pyruvate carboxylase
PIP: Plasma membrane intrinsic protein
RFO: Raffinose family of oligosaccharides
RH: Relative humidity
RMA: Robust multi-array average
ROS: Reactive oxygen species
RWC: Relative water content
SDAT: Significantly differentially abundant transcripts
TCA: Tricarboxylic acid
TF: Transcription factor
TIP: Tonoplast membrane intrinsic protein
