# Supplementary figures and images for: Sporobolus stapfianus: Insights into desiccation tolerance in the resurrection grasses from linking transcriptomics to metabolomics
Source: BMC Plant Biol. 2017 Mar 28;17:67. doi: 10.1186/s12870-017-1013-7 (PMC5371216; doi:10.1186/s12870-017-1013-7)

Supplemental Figure 1

A

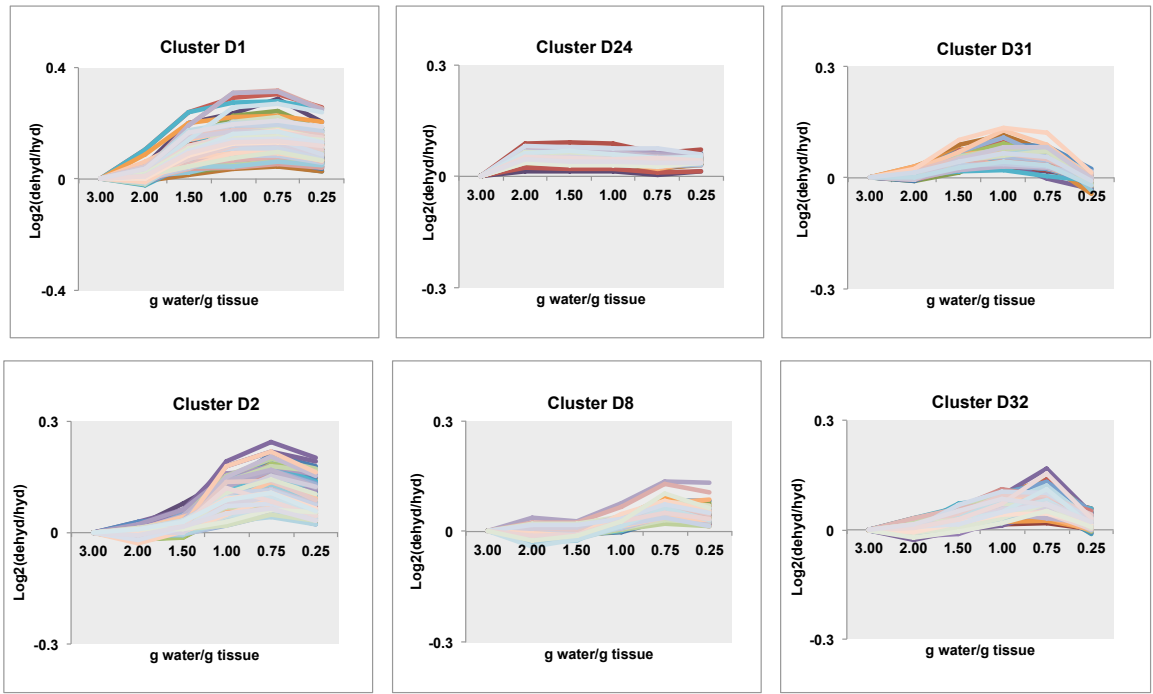

B

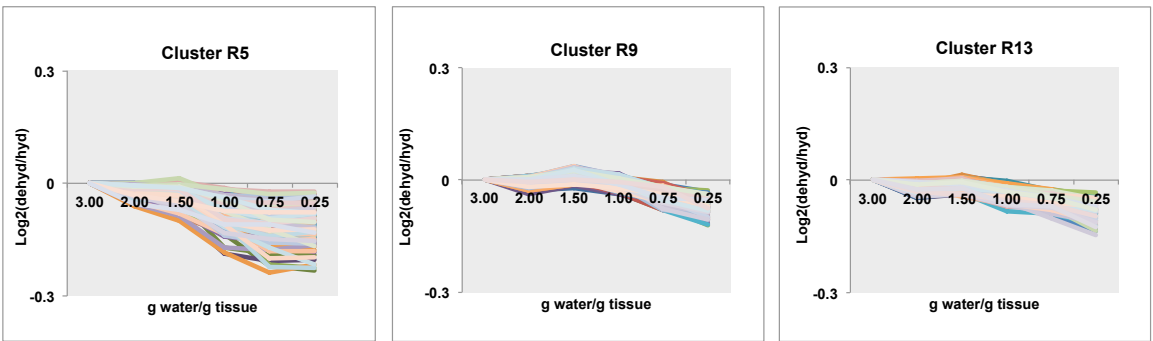

Supplement: Supplementary file 7 — Predominant clusters of SDATs that share distinct patterns of abundance during dehydration: A. Predominant patterns of abundance for transcripts in clusters that exhibited increased abundance during dehydration. B. Predominant patterns of abundance for transcripts in clusters that exhibited a decreased abundance during dehydration. (PDF 226 kb) [file 12870_2017_1013_MOESM7_ESM.pdf]
